# Association of purine asymmetry, strand-biased gene distribution and PolC within Firmicutes and beyond: a new appraisal

**DOI:** 10.1186/1471-2164-15-430

**Published:** 2014-06-04

**Authors:** Sanjoy Kumar Saha, Aranyak Goswami, Chitra Dutta

**Affiliations:** Structural Biology & Bioinformatics Division, CSIR- Indian Institute of Chemical Biology, 4, Raja S. C. Mullick Road, Kolkata, 700032 India

**Keywords:** Fusobacteria, Tenericutes, Thermotogae, G-dominance, Leading strand, Lagging strand, Mutational bias, Cytosine methylation, Codon sites, Base usage

## Abstract

**Background:**

The Firmicutes often possess three conspicuous genome features: marked Purine Asymmetry (PAS) across two strands of replication, Strand-biased Gene Distribution (SGD) and presence of two isoforms of DNA polymerase III alpha subunit, PolC and DnaE. Despite considerable research efforts, it is not clear whether the co-existence of PAS, PolC and/or SGD is an essential and exclusive characteristic of the Firmicutes. The nature of correlations, if any, between these three features within and beyond the lineages of Firmicutes has also remained elusive. The present study has been designed to address these issues.

**Results:**

A large-scale analysis of diverse bacterial genomes indicates that PAS, PolC and SGD are neither essential nor exclusive features of the Firmicutes. PolC prevails in four bacterial phyla: Firmicutes, Fusobacteria, Tenericutes and Thermotogae, while PAS occurs only in subsets of Firmicutes, Fusobacteria and Tenericutes. There are five major compositional trends in Firmicutes: (I) an explicit PAS or G + A-dominance along the entire leading strand (II) only G-dominance in the leading strand, (III) alternate stretches of purine-rich and pyrimidine-rich sequences, (IV) G + T dominance along the leading strand, and (V) no identifiable patterns in base usage. Presence of strong SGD has been observed not only in genomes having PAS, but also in genomes with G-dominance along their leading strands – an observation that defies the notion of co-occurrence of PAS and SGD in Firmicutes. The PolC-containing non-Firmicutes organisms often have alternate stretches of R-dominant and Y-dominant sequences along their genomes and most of them show relatively weak, but significant SGD. Firmicutes having G + A-dominance or G-dominance along LeS usually show distinct base usage patterns in three codon sites of genes. Probable molecular mechanisms that might have incurred such usage patterns have been proposed.

**Conclusion:**

Co-occurrence of PAS, strong SGD and PolC should not be regarded as a genome signature of the Firmicutes. Presence of PAS in a species may warrant PolC and strong SGD, but PolC and/or SGD not necessarily implies PAS.

**Electronic supplementary material:**

The online version of this article (doi:10.1186/1471-2164-15-430) contains supplementary material, which is available to authorized users.

## Background

Three conspicuous genome features often co-occur in the Firmicutes. These are: (i) a pronounced Purine Asymmetry (PAS) with the dominance of purine bases (R = G/A) over pyrimidines (Y = C/T) along the entire leading strand of replication [1, 2], (ii) a strong Strand-specific bias in Gene Distribution (SGD), i.e., the presence of significantly larger population of genes, especially the essential and highly expressed ones, in the leading strand (LeS), as compared to that in the respective lagging strand (LaS) [3–5] and (iii) presence of two different isoforms of DNA polymerase III (PolIII) alpha subunit, PolC and DnaE, that are responsible for the synthesis of the LeS and LaS respectively [1, 3, 6]. Among these, the feature of SGD is not limited to the Firmicutes only. It exists in a large number of bacteria from diverse lineages, but the bias is the strongest in Firmicutes [3], reaching even 87% in some of its members such as *Thermoanaerobacter tengcongensis*[1, 7].

The other two genome features, PAS and PolC are believed to be the signature of the Firmicutes only [1]. Some stray cases of the existence of PolC in Fusobacteria and Themotogae were reported earlier [8], but these were taken as putative outcome of lateral gene transfer. Existence of PAS or G + A-dominance in LeS in any non-Firmicutes species is yet to be reported, though dominance of guanine along LeS is a common trait in bacteria [3, 9]. Earlier studies on Firmicutes attributed PAS to several factors [1, 10–13]. A selection pressure exerted by PolC is believed to be the major contributor [11, 13]. Other plausible factors that might be responsible for PAS include an affinity in the genes to be co-oriented with the replicating fork [12], selective avoidance of stop codons and underrepresentation of costly amino acids [10]. A correlation between PAS and SGD might also exist [1]. It is worth mentioning at this point that a different type of strand-specific compositional bias - an enrichment of guanine and thymine (G + T) in the LeSs – has earlier been observed in many non-Firmicutes bacterial species [14–16]. This trait, which is more frequent among the strictly host-associated endosymbionts or pathogens with reduced genomes [17–20], has been attributed to the strand-biased deamination and 5-methylation of cytosine [9, 21].

All the studies on PAS, PolC and SGD reported so far, however, suffer from certain limitations. Some of these reports were based on limited number of genomes. For instance, the study proposing potential correlations between PAS, PolC and SGD [1] relied on a comparative analysis of only two model examples of Firmicutes and non-Firmicutes - *Bacillus anthracis str. Ames 0581* and *Francisella tularensis* respectively. One may, however, argue whether the observations made in the study should be extrapolated to the entire bacterial kingdom or not. There were some large scale studies on strand-specific asymmetries in nucleotide composition and gene distribution in Firmicutes, which focused on the average biases in sequence composition at the whole genome levels [2, 12, 13]. However, none of these studies mentioned whether such global asymmetries also persist locally at smaller scales along the LeS or LaS of the respective genomes. There was also an effort towards the analysis of inter-strand variations in amino acid and codon usage in three DnaE-based groups of bacteria [2], but it focused only on the overall compositional features of those three groups. Additionally, the study did not pay attention to the preservence of the three features - PolC, PAS and SGD across the members within a group, especially when they thrive at diverse ecological conditions.

Studies on the Firmicutes, therefore, have left some pertinent questions unaddressed. Is PAS or G + A-dominance really an essential as well as exclusive feature of the Firmicutes? Do the usages of both guanine and adenine individually contribute to PAS across the whole genomes of Firmicutes species? Does the trait of PAS persist at local levels along all the LeS sequences of the Firmicutes? If yes, how does it influence the nucleotide usages in synonymous and non-synonymous codon sites of genes? Do PAS, PolC or SGD always co-occur in a bacterial genome? If not, how do they correlate with one another? In an attempt to address all these enigmatic issues, we have examined the status of PAS, SGD & PolC in diverse bacterial species (selected in a way to cover different genera of the phylum Firmicutes as well as other non-Firmicutes phyla of the bacterial world).

Our analysis reveals that co-existence of PAS, PolC and SGD is neither exclusive nor essential signature of the Firmicutes. These features co-exist only in a subset of the Firmicutes and also occur, either collectively or individually, in members of three other bacterial phyla - Fusobacteria, Tenericutes and Thermotogae. Almost all Firmicutes species contain PolC, but the usage of guanine and that of adenine do not always contribute individually to PAS across their whole genomes. A large number of Firmicutes members show the dominance of only guanine, but not of adenine, along their LeSs. Existence of some other trends like G + T dominance along LeS or presence of alternate segments of R and Y rich sequences along the genomes have also been observed. The study indicates that PAS might assure the presence of PolC and SGD, but the reverse is not true.

## Results

### PAS is neither an exclusive nor an essential feature of the Firmicutes

**Figure 1:**
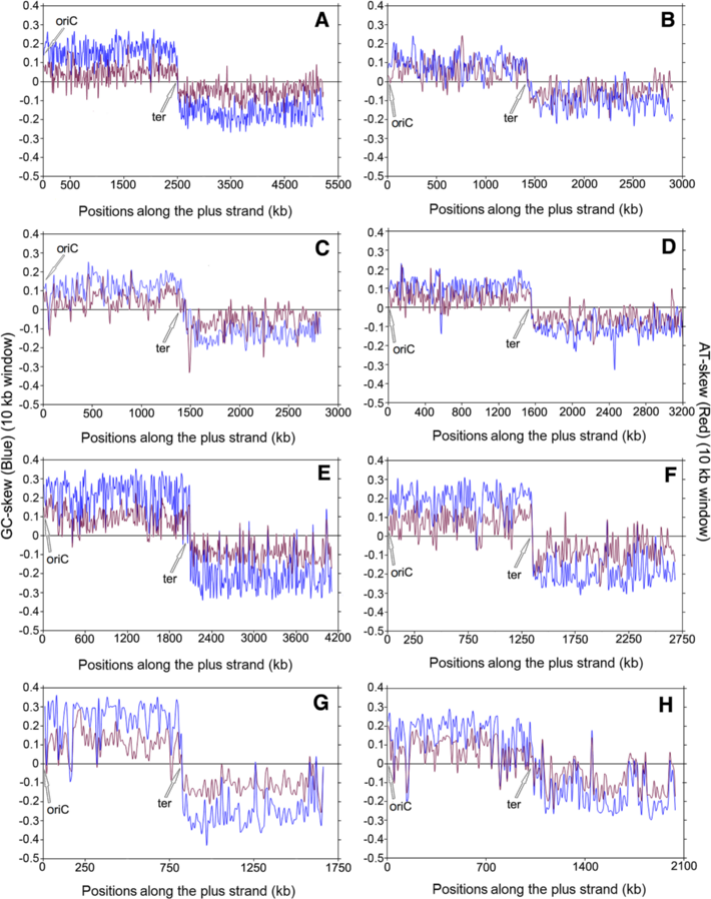
**Instantaneous GC-skew (blue lines) and AT-skew (red lines) trajectories in model representatives of Trend I. (A)** *Bacillus anthracis str.Ames*, **(B)** *Listeria monocytogenes 07PF0776*, **(C)** *Staphylococcus aureus 04–02981*, **(D)** *Enterococcus faecalis V583*, **(E)** *Clostridium difficile CD196*, **(F)** *Thermoanaerobacter tengcongensis MB4*, **(G)** *Streptobacillus moniliformis DSM 12112*, **(H)** *Illyobacter polytropus DSM 2926*.

**Figure 2:**
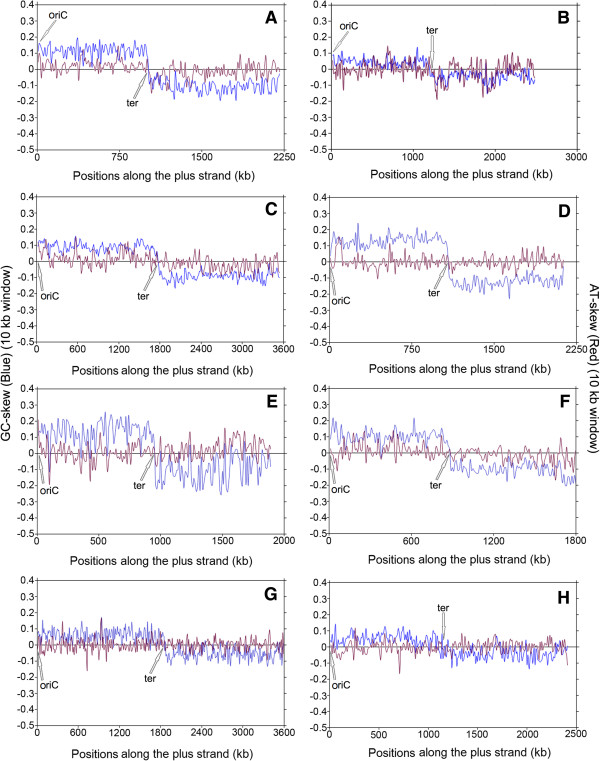
**Instantaneous GC-skew (blue lines) and AT-skew (red lines) trajectories in model representatives of Trend II. (A)** *Streptococcus agalactiae NEM 316*, **(B)** *Acidaminococcus intestini RyC-MR95*, **(C)** *Geobacillus kaustophilus HTA426*, **(D)** *Veillonella parvula DSM 2008*, **(E)** *Thermodesulfobium narugense DSM 14796*, **(F)** *Clostridiales genomosp BVAB3 UPII9 5*, **(G)** *Acinetobacter sp. ADP1*, **(H)** *Candidatus Protochlamydia amoebophila UWE25*.

**Figure 3:**
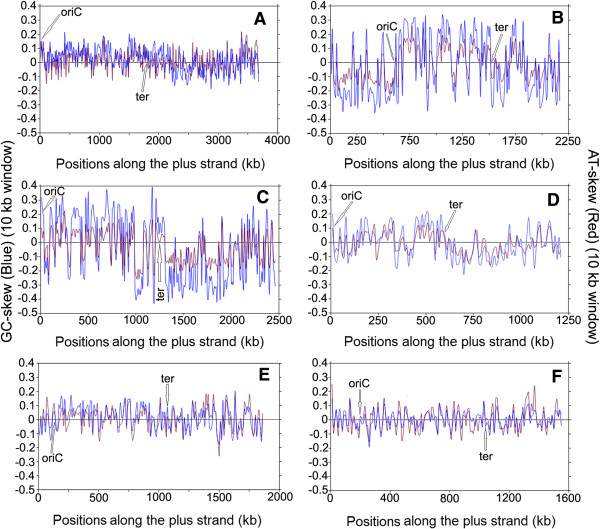
**Instantaneous GC-skew (blue lines) and AT-skew (red lines) trajectories in model representatives of Trend III. (A)** *Ruminocococcus albus 7*, **(B)** *Fusobacterium nucleatum subsp. nucleatum ATCC 25586*, **(C)** *Leptotrichia buccalis C-1013-b*, **(D)** *Mycoplasma mycoides SC PG1*, **(E)** *Thermotoga maritima MSB8,* **(F)** *Aquifex aeolicus VF5*.

**Figure 4:**
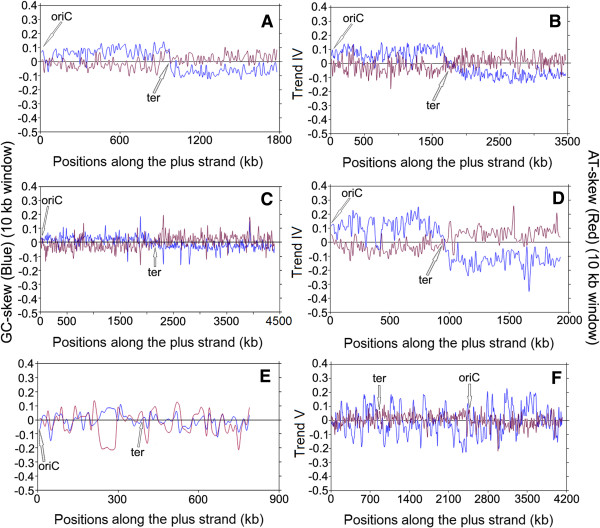
**Instantaneous GC-skew (blue lines) and AT-skew (red lines) trajectories in model representatives of Trend IV & V.** Trend IV - **(A)** *Oenococcus oeni PSU1*, **(B)** *Sulfobacillus acidophilus DSM 10332*, **(C)** *Mycobacterium tuberculosis CDC 1511*, **(D)** *Bartonella henselae str. Houston-1.* Trend V - **(E)** *Mycoplasma synoviae 53*, **(F)** *Acidobacterium capsulatum ATCC 51196.*

**Figure 5:**
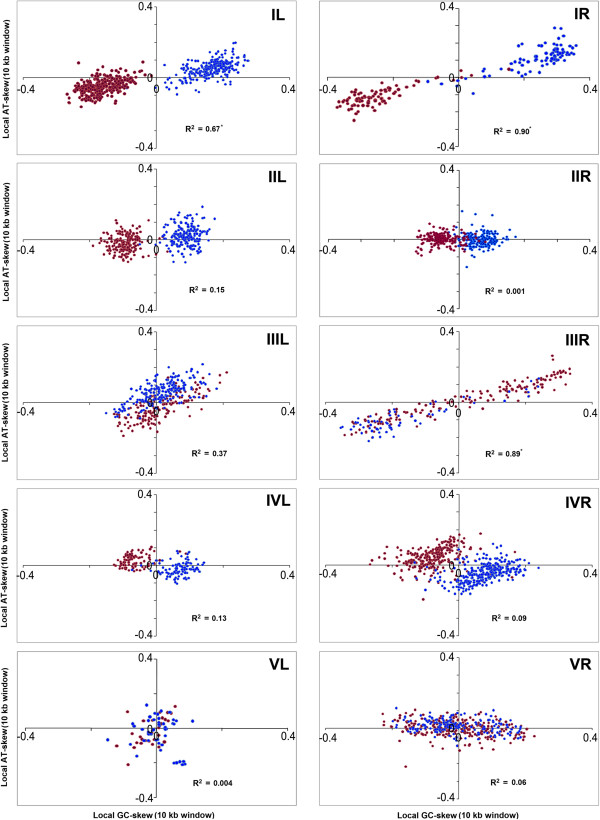
**Scatter plots of Local GC-skew and AT-skew values in model representatives of organisms following different trends in purine usages. (I)** Trend I - *Bacillus anthracis str.Ames* (L) and *Streptobacillus moniliformis DSM 12112* (R); **(II)** Trend II - *Geobacillus kaustophilus HTA 426* (L) and *Acinetobacter sp. ADP1* (R); **(III)** Trend III - *Ruminocococcus albus 7* (L) and *Fusobacterium nucleatum subsp.nucleatum ATCC 25586* (R); **(IV)** Trend IV - *Oenococcus oeni PSU1* (L) and *Bacteroides fragilis 638R* (R); **(V)** Trend V - *Mycoplasma synoviae 53* (L) and *Acidobacterium capsulatum ATCC 51196* (R).

With a view to examine the status of PAS within and beyond the Firmicutes lineage, variations in local GC-skew and AT-skew values (averaged over 10 kb segments along the plus strands of the respective genomes) were studied in each of the organisms under study (Additional file 1: Table S1 and Additional file 2: Table S2). These skew trajectories may be classified into five distinct trends, as described in the Methods section. Some model examples of these five different trends in skew trajectories have been presented in Figures 1, 2, 3 and 4. In order to rule out any ambiguity while identifying such trends in skew trajectories, we have also examined the scatter plots of the local GC-skew and AT-skew values for each species under study. Some representative examples of such scatter plots are shown in Figure 5I-V.

#### Trend I – Explicit PAS with individual dominance of G and A along the entire LeS

Trend I refers to the cases, where both the purine bases (guanine and adenine) individually contribute to the purine-richness of the LeSs. Some typical examples of Trend I species are shown in Figure 1A–H, where local GC-skew and AT-skew values are, by and large, positive between the putative origin (oriC) and termination (ter) sites of replication along the plus strand, and negative in the other half of the genomes; with a sharp transition from the positive to negative values at ter (Figure 1). In most of the Trend I organisms, more than 70% of the 10 kb LeS segments have exceptionally high frequencies of both guanine and adenine as compared to cytosine and thymine respectively (Tables 1 and 2), while the number of LeS segments of other three possible combinations (b), (c) or (d) are significantly low in most cases. These observations indicate that the LeS sequences have explicit enrichment of both the purine bases (guanine and adenine) in all the organisms of Trend I. We shall henceforth refer to this trend as explicit PAS or simply PAS.

**Table 1:** **Status of combinations (a) – (d), PAS, SGD and PolC in Firmicutes taken in this study**

Organisms	% of 10 kb segments along LeS with combinations^@^				PAS	SGD		PolC (Y/N)
	(a)	(b)	(c)	(d)				
	G > C	G > C	G ≤ C	G ≤ C		LeS	p^	
	A > T	A ≤ T	A > T	A ≤ T				
**Trend I**								
*A. woodii*	**90.1**	8.4	0.5	1	Y	0.78	***	Y
*A. fermentans*	**89.2**	7.3	3	0.4	Y	0.84	***	Y
*A. arabaticum*	**87.8**	11.8	0	0.4	Y	0.9	***	Y
*A. urinae*	**69.7**	*29.3*	1	0	Y	0.79	***	Y
*A. metalliredigens*	**96.5**	2.2	0.8	0.4	Y	0.86	***	Y
*A. prevotii*	**88.8**	9.6	0.5	1.1	Y	0.84	***	Y
*A. flavithermus*	**73.9**	*23.6*	1.4	1.1	Y	0.73	***	Y
*B. amyloliquefaciens*	**82.9**	15.1	0.5	1.5	Y	0.74	***	Y
*B. anthracis*	**87.2**	12.3	0	0.6	Y	0.75	***	Y
*B. atrophaeus*	**78.8**	16.6	0.7	3.9	Y	0.74	***	Y
*B. cellulosilyticus*	**86.7**	9.9	1.5	1.9	Y	0.77	***	Y
*B. cereus*	**86.9**	11.9	0	1.2	Y	0.73	***	Y
*B. clausii*	**72.6**	*26.5*	0.5	0.5	Y	0.76	***	Y
*B. cytotoxicus*	**87.5**	12	0	0.5	Y	0.75	***	Y
*B. halodurans*	**77.4**	*20.5*	0.7	1.4	Y	0.77	***	Y
*B. licheniformis*	**79.9**	18.3	0.9	1	Y	0.74	***	Y
*B. megaterium*	**89.6**	9	1.4	0	Y	0.75	***	Y
*B. pseudofirmus*	**84.7**	13.8	1	0.5	Y	0.77	***	Y
*B. pumilus*	**84.3**	13	1.9	0.8	Y	0.75	***	Y
*B. selenitireducens*	**71.3**	*27*	0.6	1.1	Y	0.76	***	Y
*B. subtilis*	**80.3**	17.3	0.7	1.7	Y	0.74	***	Y
*B. thuringiensis*	**87.9**	11.9	0	0.2	Y	0.75	***	Y
*B. weihenstephanensis*	**86.9**	12.2	0.2	0.8	Y	0.73	***	Y
*B. brevis*	**82.2**	16.9	0.5	0.5	Y	0.74	***	Y
*B. proteoclasticus*	**85.4**	14.7	0	0	Y	0.86	***	Y
*C. bescii*	**88.3**	5.8	0.7	5.2	Y	0.81	***	Y
*C. hydrothermalis*	**87.7**	8.3	0.7	3.3	Y	0.81	***	Y
*C. hydrogenoformans*	**84.6**	15.4	0	0	Y	0.87	***	Y
*C. saccharolyticus*	**87.8**	7.1	1.4	3.7	Y	0.81	***	Y
*C. sp.*	**94.3**	4.6	0.4	0.8	Y	0.78	***	Y
*C. acetobutylicum*	**91.6**	5.6	0.3	2.5	Y	0.79	***	Y
*C. autoethanogenum*	**87.1**	8.5	0.7	3.7	Y	0.77	***	Y
*C. beijerinckii*	**96.8**	2	0	1.2	Y	0.83	***	Y
*C. botulinum*	**95.9**	2.8	0	1.3	Y	0.82	***	Y
*C. cellulovorans*	**92.2**	6.1	0.4	1.3	Y	0.8	***	Y
*C. difficile*	**92**	4.6	0.5	2.9	Y	0.81	***	Y
*C. lentocellum*	**91.8**	4.2	0.7	3.3	Y	0.84	***	Y
*C. sticklandii*	**95.6**	3	0.7	0.7	Y	0.83	***	Y
*C. novyi*	**96.1**	3.15	0	0.8	Y	0.84	***	Y
*D. reducens*	**81.9**	14.7	1.4	1.9	Y	0.8	***	Y
*E. faecalis*	**89.4**	9.4	0.6	0.6	Y	0.8	***	Y
*E. faecium*	**91**	9.0	0	0	Y	0.71	***	Y
*E. rhusiopathiae*	**71.9**	1.1	*25.8*	1.1	Y	0.79	***	Y
*E. rectale*	**94.8**	2.0	0	3.2	Y	0.82	***	Y
*E. AT1b*	**78.9**	*20.7*	0	0.3	Y	0.64	***	Y
*E. sibiricum*	**84.5**	14.5	0.3	0.7	Y	0.7	***	Y
*F. magna*	**89.9**	5.6	1.7	2.8	Y	0.83	***	Y
*H. hydrogeniformans*	**91.6**	6.1	0.8	1.5	Y	0.89	***	Y
*H. halophilus*	**78.1**	19.3	0.2	2.4	Y	0.74	***	Y
*L. acidophilus*	**73.9**	*25.6*	0	0.5	Y	0.74	***	Y
*L. amylovorus*	**74.3**	*25.2*	0.5	0.0	Y	0.75	***	Y
*L. gasseri*	**79.9**	19.1	0.5	0.5	Y	0.77	***	Y
*L. garvieae*	**81.6**	15.8	0.5	2	Y	0.78	***	Y
*L. lactis cremoris*	**80.1**	18.7	0.4	0.8	Y	0.8	***	Y
*L. lactis lactis*	**85.7**	11.6	1.6	1.2	Y	0.81	***	Y
*L. mesenteroides*	**78.8**	*20.7*	0.5	0	Y	0.83	***	Y
*L. innocua*	**84.4**	11	3.7	1	Y	0.8	***	Y
*L. monocytogenes*	**83.5**	11.4	4.8	0.3	Y	0.79	***	Y
*L. seeligeri*	**86.4**	10	2.9	0.7	Y	0.79	***	Y
*L. sphaericus*	**80.1**	17.1	0.9	2	Y	0.74	***	Y
*N. thermophilus*	**81**	15.8	0.3	2.9	Y	0.8	***	Y
*O. iheyensis*	**84**	13.2	0.8	1.9	Y	0.75	***	Y
*O. valericigenes*	**52.1**	23	10	15	Y	0.61	***	Y
*S. ruminantium*	**70.5**	*29.5*	0	0	Y	0.86	***	Y
*P. Y412MC10*	**76.4**	*22.8*	0.4	0.4	Y	0.77	***	Y
*R. hominis*	**97.5**	2.2	0	0.3	Y	0.87	***	Y
*S. silvestris*	**86.9**	9.3	1.5	2.3	Y	0.76	***	Y
*S. aureus*	**86.1**	11.4	1.1	1.4	Y	0.75	***	Y
*S. epidermidis*	**83.1**	14.1	1.2	1.6	Y	0.73	***	Y
*S. haemolyticus*	**83.2**	13.5	1.9	1.5	Y	0.74	***	Y
*S. lugdunensis*	**80.8**	15.1	1.9	2.3	Y	0.74	***	Y
*S. lipocalidus*	**74.4**	*21.4*	1.7	2.5	Y	0.8	***	Y
*S. wolfei*	**76.5**	16	4.1	3.4	Y	0.78	***	Y
*T. acetatoxydans*	**92.4**	3.3	2.9	1.5	Y	0.84	***	Y
*T. pseudethanolicus*	**92.4**	6.8	0	0.9	Y	0.87	***	Y
*T. tengcongensis*	**87.3**	11.2	0.4	1.1	Y	0.86	***	Y
**Trend II**								
*A. intestini*	**54.3**	**40.5**	2.8	2.4	N	0.8	***	Y
*A. acidocaldarius*	**39.9**	**58.8**	1	0.3	N	0.78	***	Y
*A. degensii*	**45.9**	**49.3**	0	4.7	N	0.82	***	Y
*C. genomosp*	**63.9**	**34.4**	0	1.7	N	0.78	***	Y
*C. proteolyticus*	**44**	**52.5**	1.4	2.1	N	0.69	***	Y
*D. hafniense*	**67.3**	**30.6**	1.3	0.8	N	0.79	***	Y
*D. acetoxidans*	**58.8**	**34.6**	1.8	4.8	N	0.75	***	N
*D. ruminis*	**58.4**	**34.4**	2	5.3	N	0.77	***	Y
*E. harbinense*	**35**	**47**	6	12	N	0.58	***	Y
*G. kaustophilus*	**66.1**	**32.2**	0.3	1.4	N	0.79	***	Y
*M. thermoacetica*	**62.1**	**30.3**	3.1	4.6	N	0.81	***	Y
*P. polymyxa*	**67.1**	**30.6**	0.4	1.9	N	0.75	***	Y
*L. brevis*	**57.2**	**41.9**	0.9	0	N	0.74	***	Y
*S. sputigena*	**61.2**	**36.1**	1.2	1.6	N	0.8	***	Y
*S. agalactiae*	**65.2**	**34.8**	0	0	N	0.82	***	Y
*S. equi*	**39.3**	**58.9**	0.5	1.4	N	0.81	***	Y
*S. pneumoniae*	**59.8**	**38.7**	0	1.5	N	0.8	***	Y
*S. pyogenes*	**62.7**	**35.1**	1.6	0.5	N	0.79	***	Y
*S. thermophilum*	**46.9**	**47.5**	3.4	2.3	N	0.73	***	Y
*T. marianensis*	**41.8**	**53.2**	3.6	1.4	N	0.76	***	N
*T. narugense*	**43.4**	**47.6**	0	9	N	0.72	***	Y
*V. parvula*	**46.7**	**52.9**	0	0.5	N	0.88	***	Y
**Trend III**								
*R. albus*	**52.5**	18.2	7.6	*21.7*	N	0.6	***	Y
**Trend IV**								
*B. tusciae*	*26.4*	**72**	0.5	1.1	N	0.69	***	Y
*O. oeni*	*26.6*	**69.5**	1.1	2.8	N	0.74	***	Y
*S. acidophilus*	*25.7*	**70.6**	2.9	0.9	N	0.71	***	Y

^@^Bolds are significant at p < 0.05, italics are random.

^p value: *** <0.001.

**Table 2:** **Status of combinations (a) – (d), PAS, SGD and PolC in the non-Firmicutes organisms examined in this study**

Organisms	Taxonomy	% of 10 kb segments along LeS with combinations^@^				PAS	SGD		PolC (Y/N)
		(a)	(b)	(c)	(d)				
		G > C	G > C	G ≤ C	G ≤ C		LeS	p^	
		A > T	A ≤ T	A > T	A ≤ T				
**Trend I**									
*I. polytropus*	Fusobacteria	**82.8**	5.9	1.5	9.8	Y	0.76	***	Y
*S. termitidis*		**89.3**	4.1	0.7	5.9	Y	0.73	***	Y
*S. moniliformis*		**92.8**	1.8	0.6	4.8	Y	0.84	***	Y
*A. laidlawii*	Tenericutes	**94.6**	4.0	0.7	0.7	Y	0.87	***	Y
*M. florum*		**94.9**	1.3	2.5	1.3	Y	0.89	***	Y
*M. gallisepticum*		**72.9**	8.3	13.5	5.2	Y	0.77	***	Y
*U. parvum*		**66.7**	13.3	5.3	14.7	Y	0.6	***	Y
*U. urealyticum*		**66.7**	10.3	6.9	16.1	Y	0.67	***	Y
**Trend II**									
*S. meliloti*	Alphaproteobacteria	**35.3**	**44.9**	5.2	14.6	N	0.56	***	N
*A. aromaticum*	Betaproteobacteria	**33.4**	**51.4**	7.9	7.2	N	0.56	***	N
*B. thetaiotaomicron*	Bacteroidetes/ Chlorobi	**30.2**	**60.5**	2.4	6.9	N	0.52	NS	N
*P. gingivalis*		**31.5**	**38.8**	11.6	18.1	N	0.54	*	N
*S. ruber*		**52.0**	**40.4**	5.1	2.5	N	0.57	***	N
*C. protochlamydia*	Chlamydiae/ Verrucomicrobia	**35.3**	**51.5**	6.2	7.1	N	0.51	NS	N
*C. trachomatis*		**35.9**	**63.1**	0.0	1.0	N	0.52	NS	N
*T. thermophilus*	Deinococcus-Thermus	**30.7**	**53.4**	2.7	13.2	N	0.51	NS	N
*S. aciditrophicus*	Deltaproteobacteria	**44.5**	**38.8**	3.2	13.6	N	0.56	***	N
*E. minutum*	Elusimicrobia	**53.7**	**42.1**	0.6	3.7	N	0.65	***	N
*C. jejuni*	Epsilonproteobacteria	**47.0**	**39.0**	3.1	11.0	N	0.6	***	N
*H. hepaticus*		**40.8**	**48.0**	1.7	9.5	N	0.57	***	N
*W. succinogenes*		**31.3**	**66.7**	0.0	2.0	N	0.59	***	N
*A. sp.*	Gammaproteobacteria	**43.2**	**49.0**	4.2	3.6	N	0.59	***	N
*E. coli*		**38.2**	**49.5**	7.1	5.2	N	0.55	**	N
*F. tularensis*		**45.2**	**43.6**	2.7	8.5	N	0.6	***	N
*H. ducreyi*		**35.5**	**52.1**	4.7	7.7	N	0.6	***	N
*D. acetiphilus*	Other Bacteria	*29.8*	**57.5**	3.7	9.0	N	0.56	***	N
*L. borgpetersenii*	Spirochaetes	**33.2**	**57.6**	4.5	4.8	N	0.56	***	N
*T. denticola*		**39.6**	**40.6**	3.2	16.6	N	0.55	***	N
**Trend III**									
*W. endosymbiont*	Alphaproteobacteria	**34.9**	12.7	7.9	**44.4**	N	0.53	NS	N
*A. aeolicus*	Aquificae	**42.6**	11.0	9.0	**37.4**	N	0.52	NS	N
*H. Y04AAS1*		**36.8**	13.6	16.1	**33.6**	N	0.52	NS	N
*P. marina*		**37.3**	12.4	7.3	**43.0**	N	0.52	NS	N
*S. YO3AOP1*		**45.6**	3.3	9.9	**41.2**	N	0.56	***	N
*F. nucleatum*	Fusobacteria	**71.9**	2.3	2.3	*23.5*	N	0.58	***	Y
*L. buccalis*		**68.3**	2.9	2.0	*26.8*	N	0.6	***	Y
*M. capricolum*	Tenericutes	**66.3**	8.9	0.0	*24.8*	N	0.7	***	Y
*M. mobile*		**52.6**	9.2	15.8	*22.4*	N	0.57	**	Y
*M. mycoides*		**59.7**	5.0	9.2	*26.1*	N	0.63	***	Y
*M. pulmonis*		**54.7**	13.7	7.4	*24.2*	N	0.62	***	Y
*F. nodosum*	Thermotogae	**36.6**	6.2	17.0	**40.2**	N	0.53	NS	Y
*K. olearia*		**32.5**	3.5	*24.6*	**39.5**	N	0.54	*	Y
*P. mobilis*		**44.9**	13.9	6.5	**34.7**	N	0.53	NS	Y
*T. africanus*		**34.3**	9.0	*24.9*	**31.8**	N	0.56	***	Y
*T. maritima*		**48.7**	13.5	7.0	**30.8**	N	0.5	NS	Y
*T. naphthophila*		**45.0**	13.9	6.1	**35.0**	N	0.52	NS	Y
**Trend IV**									
*L. xyli*	Actinobacteria	17.4	**45.0**	13.6	*24.0*	N	0.61	***	N
*M. tuberculosis*		22.1	**63.6**	5.5	8.9	N	0.58	***	N
*S. coelicolor*		18.6	**47.2**	19.9	14.3	N	0.55	***	N
*A. phagocytophilum*	Alphaproteobacteria	14.3	**64.0**	11.6	10.2	N	0.58	***	N
*B. henselae*		15.0	**82.4**	0.0	2.6	N	0.58	***	N
*N. sennetsu*		4.7	**83.5**	1.2	10.6	N	0.59	***	N
*Z. mobilis*		14.9	**60.9**	12.6	11.6	N	0.56	**	N
*C. tepidum*	Bacteroidetes/ Chlorobi	14.4	**79.1**	2.8	3.7	N	0.55	***	N
*B. bronchiseptica*	Betaproteobacteria	*27.1*	**64.7**	4.7	3.6	N	0.55	***	N
*N. meningitidis*		*29.2*	**55.3**	8.9	6.6	N	0.54	**	N
*N. europaea*		*22.9*	**69.2**	3.2	4.7	N	0.51	NS	N
*P. necessarius*		*20.5*	**77.2**	0.0	2.3	N	0.62	***	N
*R. solanacearum*		*28.9*	**53.4**	11.2	6.5	N	0.59	***	N
*C. caviae*	Chlamydiae/ Verrucomicrobia	15.5	**78.5**	0.9	5.2	N	0.52	NS	N
*W. chondrophila*		18.0	**78.7**	1.0	2.4	N	0.51	NS	N
*C. aggregans*	Chloroflexi	15.3	**62.4**	12.0	10.3	N	0.53	*	N
*D. CBDB1*		15.1	**73.4**	3.6	7.9	N	0.52	NS	N
*M. ruber*	Deinococcus- Thermus	*26.1*	**59.3**	6.8	7.8	N	0.54	**	N
*B. bacteriovorus*	Deltaproteobacteria	*26.4*	**72.0**	0.5	1.1	N	0.56	***	N
*D. psychrophila*		9.4	**84.7**	1.7	4.3	N	0.53	*	N
*G. sulfurreducens*		*22.6*	**54.2**	10.8	12.4	N	0.64	***	N
*L. intracellularis*		17.2	**77.2**	0.7	4.8	N	0.5	NS	N
*A. vinelandii*	Gammaproteobacteria	15.3	**66.7**	8.2	9.7	N	0.56	***	N
*S. amazonensis*		19.2	**77.8**	0.9	2.1	N	0.56	***	N
*X. fastidiosa*		1.1	**81.3**	12.0	5.6	N	0.57	***	N
*P. limnophilus*	Planctomycetes	*20.8*	**48.0**	15.2	16.0	N	0.5	NS	N
*R. baltica*		13.2	**57.4**	*23.0*	6.4	N	0.51	NS	N
*B. burgdorferi*	Spirochaetes	11.0	**87.9**	1.1	0.0	N	0.66	***	N
*S. smaragdinae*		16.6	**69.7**	0.7	13.1	N	0.63	***	N
**Trend V**									
*A. capsulatum*	Acidobacteria	16.4	**38.1**	*21.3*	*24.2*	N	0.5	NS	N
*C. Solibacter*		*28.0*	*24.2*	*21.8*	*26.0*	N	0.53	**	N
*B. longum*	Actinobacteria	*20.5*	**32.6**	**38.4**	8.5	N	0.54	**	N
*N. farcinica*		*22.3*	**41.3**	*21.3*	15.1	N	0.57	***	N
*C. atlanticus*	Bacteroidetes/ Chlorobi	**47.8**	*20.5*	2.7	*29.0*	N	0.51	NS	N
*R. RS 1*	Chloroflexi	17.9	**41.2**	*26.0*	14.8	N	0.51	NS	N
*C. sp.*	Cyanobacteria	*24.3*	*20.8*	*28.4*	*26.5*	N	0.51	NS	N
*N. sp.*		*23.8*	*29.1*	*25.5*	*21.6*	N	0.5	NS	N
*P. marinus*		6.0	**71.4**	0.0	*22.6*	N	0.52	NS	N
*T. erythraeum*		**32.9**	19.5	19.2	*28.4*	N	0.51	NS	N
*D. geothermalis*	Deinococcus- Thermus	16.7	**48.4**	11.8	*23.2*	N	0.51	NS	N
*H. pylori*	Epsilonproteobacteria	**45.5**	*23.6*	8.5	*22.4*	N	0.52	NS	N
*C. Phytoplasma*	Tenericutes	*22.7*	**30.7**	*28.4*	18.2	N	0.56	*	Y
*M. synoviae*		**40.5**	**20.3**	13.9	*25.3*	N	0.5	NS	Y
*O. yellows*		12.9	**42.4**	**41.2**	3.5	N	0.64	***	Y
*T. lettingae*	Thermotoga	**37.6**	**30.1**	4.2	*28.2*	N	0.51	NS	Y

^@:^ Bolds are significant at P < 0.05, italics are random.

^: p value ranges are: NS > 0.05, * <0.05, ** <0.01, *** <0.001.

Presence of Trend I are found in more than 70% of the Firmicutes under study and it is predominant among the members of *Bacillales*, especially in those belonging to the genera of *Bacillus, Listeria*, *Staphylococcus*, *Enterococcus* and *Thermoanaerobcter* (Figure 1A-D, F). However, *Bacillus* is the only genus among Firmicutes, all members of which show predominance of both guanine and adenine along the LeS. Trend I has been observed in some members of *Clostridia* also (Figure 1E).

Interestingly enough, Trend I is not confined to the lineage of Firmicutes only. It has also been observed in some Fusobacteria and Tennericutes. Of the five Fusobacteria and twelve Tenericutes species studied (Additional file 2: Table S2), three Fusobacteria including *S. moniliformis* (Figure 1G)*, I. polytropus* (Figure 1H) and five Tenericutes (Table 2) display explicit PAS, indicating that PAS is not an exclusive characteristic of Firmicutes only.

None of the non-Firmicutes, non-Fusobacteria and non-Tenericutes organisms under study exhibited unequivocal G + A-enrichment of LeS. It suggests that the presence of PAS might be confined only to the three bacterial phyla, Firmicutes, Fusobacteria and Tenericutes, which are thought to be closely related from the evolutionary point of view [8].

#### Trend II – Only G-dominance in LeS with no unequivocal trend in adenine usage

All Firmicutes genera except *Bacillus* include certain members, which show dominance of only guanine, but not of adenine along the LeS. This trend (Trend II) has also been observed in a number of non-Firmicutes species from diverse bacterial phyla. Some model examples of Trend II have been depicted in Figure 2, where the representatives of Firmicutes are shown in Figures 2A-F, and those of non-Firmicutes in Figure 2G-H. In all cases, the GC-skew trajectory exhibits a sharp transition in sign only once at the oriC/ter region, but AT-skew values undergo irregular oscillation around the null axis, showing no definite pattern. Cumulative GC and AT-skew trajectories and instantaneous RY skew values of the respective species are shown in Additional file 3: Figure S1. As expected, the cumulative GC-skew always increases between oriC and ter and decreases along the other half of the plus strand. But the nature of the cumulative AT-skew varies from species to species and in majority of the organisms following Trend II, hardly deviating from the null value (Additional file 3: Figure S1 CL, DL, EL, GL & HL). In all Firmicutes members of this category, the magnitude of GC-skew values is usually much higher than the respective AT-skew values. Hence the average local purine-content of LeS sequences remain higher than the respective pyrimidine content (Additional file 3: Figure S1), but the total contribution to such apparent purine-richness of LeS comes from the G-dominance only with little or no contribution from the adenine frequencies. However, in certain Trend II Firmicutes, the overall R- usage does not follow any definite strand-specific pattern (Additional file 3: Figure S1).

The differences between PAS (Trend I) and G-dominance (Trend II) can be clearly understood from Figure 5. In organisms having PAS (Trend I, Figure 5 IL, IR), the points from the segments between oriC and ter (blue points) usually lie in the first quadrant (barring a few exceptions). It re-confirms that both GC-skew and AT-skew values are in general positive. The points from the segments between ter and oriC (red) lie in the third quadrants indicating negative values for both the skews. On the contrary in Trend II organisms, the points corresponding to the LeS part of the plus strand are almost equally distributed in first and fourth quadrants (Figure 5, IIL, IIR, blue points), while those corresponding to the LaS parts (red points) are distributed among the second and third quadrants (red points). This indicates that the GC-skew values remain mostly positive along LeS and negative along LaS, but the AT-skew values fluctuates between positive and negative values along both the replicating strands. Fluctuations in AT-skew magnitudes along two replication strands of Trend II organisms are also apparent from Tables 1 and 2 - clearly indicating that in organisms following Trend II, frequencies of LeS segments with base usage combinations (a) and (b) both are significantly high and their values are often comparable to one another. However the presence of the other two combinations (c) and (d) are negligible, in general.

Another distinct feature of Trend I is that the pairs of instantaneous GC-skew and AT-skew values exhibit significant positive correlations for both oriC-ter (blue points) and ter-oriC (red points) regions along the plus strand (Figure 5, IL, IR). In cases of Trend II (Figure 5, IIL, IIR), no significant positive correlations exist in general between the pairs of GC-skew and AT-skew values. Even if it exists, the magnitudes of the correlation coefficients are not as high as those observed in Trend I (Figure 5, IL, IR). All these observations clearly indicate that in organisms following Trend I, usages of guanine and adenine both contribute significantly to PAS. In Trend II organisms, only an apparent purine-richness often prevails along the LeS, where the sole contribution to purine enrichment comes from the G-dominance only, with the adenine usage hardly playing any role.

Within the Firmicutes phylum, Trend II prevails in the non-*Bacilli* classes like *Clostridia* or *Negativicutes*, along with certain *Bacilli* genera like *Streptococcus*, *Geobacillus* or *Lactobacillus* etc. A small number of exceptions from the order Bacillales also fall under this category.

#### Trend III - Presence of alternate stretches of R-dominant and Y-dominant sequences along both the replicating strands

There is one Firmicutes species, *Ruminococous albus*, which exhibits a conspicuous trend of purine usage (Trend III). In this species, instantaneous GC-skew and AT-skew trajectories toggle their signs frequently and simultaneously in a way such that the respective GC and AT-skew values remain, in most cases, of the same sign (Figure 3A). Though it shows an overrepresentation of R-dominant stretches (combination (a) ≈ 53%), the Y-dominant stretches also occurs with random frequency (combination (d) ≈ 22%) [Table 1]. This suggests that a major part of the genome of *R. albus* is comprised of alternate purine-rich and pyrimidine-rich segments. A similar trend is also observed in two Fusobacteria species, namely *Fusobacterium nucleatum* (Figure 3B) and *Leptotrichia buccalis* (Figure 3C). Majority of the Tenericutes members examined in the study, including certain Mycoplasma and Ureplasma species, also follow Trend III (Figure 3D).In organisms following Trend III, local GC-skew and AT-skew values bear strong positive correlations (Figure 5, IIIL, IIIR), as observed earlier in Trend I. However, there is a major difference between the scatter plots in two trends. In Trend I, points corresponding to LeS (blue) and LaS (red) parts of the plus strands are segregated in the first and third quadrants respectively. In Trend II organisms, on the contrary, points from both the LeS and LaS sequences are distributed uniformly in the first and third quadrants, implying that both guanine and adenine frequencies are oscillating simultaneously between positive and negative values along the replicating strands.

The presence of alternate genomic segments of R-rich and Y-rich sequences was reported earlier for thermophilic/hyperthermophilic bacteria [22]. A number of thermophiles in the current dataset, especially those belonging to the Aquificae and Thermotogae lineages show the presence of Trend III in their genomes (Table 2). Two typical examples of such thermophilic organisms *Thermotoga maritima* and *Aquifex aeolicus* are presented in Figure 3E and F. The amplitudes of purine-rich/pyrimidine-rich segments of the genomes are, in general, much smaller (Figure 3A-D), but the percentage occurrence of such segments are much higher in thermophiles, as compared to the Trend III Firmicutes, Fusobacteria or Tenericutes (Tables 1 and 2). It is worth mentioning at this point that all thermophiles/hyperthermophiles does not exhibit Trend III. A substantial part of them follow a distinct trend of G + T-enrichment along LeS (Trend IV) as described below.

#### Trend IV - G + T dominance along the leading strands

In majority of the bacteria from non-Firmicutes, non-Fusobacteria, non-Tenericutes, non-Aquificae and non-Thermotogae lineages, a strand specific bias exists not in favour of G + A, but in favour of G + T usage along the entire LeS (Trend IV). Organisms following Trend IV include Proteobacteria, Actinobacteria, Bacteroides, Chloroflexi, Planctomycetes, Spirochetes etc. (Table 2). Two model examples of Trend IV genomes are shown in Figure 4(C, D), where the signs of GC-skew and AT-skew trajectories are of opposite signs. Both the skew trajectories change their signs simultaneously at oriC/ter regions, so that their LeSs have, in general, an over representation of guanine and thymine, as reported earlier [17–20]. In free living organisms, the magnitudes of the instantaneous GC-skew and AT-skew values are often quite low (Figure 4A). However, in obligatory intracellular microbes undergoing genome reduction, both GC-skew and AT-skew values are, in general, of significantly higher magnitudes confirming the general notion of their parasitic adaptation [17–20].

Though quite common among other bacteria, Trend IV is rarely seen within the Firmicutes. Among 102 Firmicutes in the dataset, only two organisms seem to follow Trend IV. These include *Oenococcus oeni -* a Lactobacillales species and *Sulfobacillus acidophilus -* a Clostridiales member (Table 1). Some typical examples of the scatter plot of local GC-skew and AT-skew values in organisms following Trend IV are shown in Figure 5 IVL and IVR. As expected, most of points from the LeS portion of the plus strand lie in the fourth quadrants (since GC-skews are positive and AT-skews are negative), but those from the LaS regions mostly appear in the second quadrants (as GC-skews are negative and AT-skews are positive, in most cases).

#### Trend V - No identifiable pattern in base usage

Lastly, there are a small number of bacterial genomes displaying random oscillation around the abscissa in both GC-skew and AT-skew trajectories. In these cases, no general trend can be detected either in the signs of GC-skew/AT-skew values or in the distribution of 10 kb segments among four combinations (a)–(d) (Table 2). Certain Tenericutes, Acidobacteria, Actinobacteria, Cyanobacteria etc. show ambiguous behavior in their GC-skew and AT-skew values (Figure 4E and F, Table 2). As expected, points in the scatter plots of GC and AT-skew values (Figure 5, VL, and VR) are also randomly distributed in all four quadrants, having no definite pattern or correlations.

### PAS, SGD and PolC might not bear any definite correlation in Firmicutes or other bacteria

As indicated in the present analysis, PAS exists in a substantial fraction of the Firmicutes but it is not a signature trait of this phylum. On the other hand, there are certain Fusobacteria and Tenericutes that clearly show the presence of PAS. In view of a recent hypothesis in favor of a correlation between PAS and SGD, it will be intriguing to examine the correspondence between PAS, PolC and SGD in Firmicutes, Fusobacteria and other organisms under study. To this end, we have checked the status of SGD as well as of PolC across all bacterial species of our dataset. Outcomes of the study are provided in Tables 1 and 2. As can be seen from these files, all organisms having PAS (Trend I) show very strong SGD. If we consider the number of 10 kb segments with G > C and A > T as a measure of the strength of PAS in a Trend I organism (Tables 1 and 2), then the scattered plot of PAS and SGD shows a strong positive correlation between themselves, the correlation coefficient being 0.59 (the scattered plot not shown).

PolC is found to be present in almost all Firmicutes members as well as in all Fusobacteria, Tenericutes and Thermotogae members under study. There are only two exceptions – *Desulfotomaculum acetoxidans* and *Thermaerobacter marianensis* both belonging to the class Clostridia under the Firmicutes phylum. *D. acetoxidans* and *T marianensis* both possess marked SGD but no PAS. BLASTP search for PolC homolog could not detect the presence of PolC in these two organisms.

All PolC-containing Firmicutes, Fusobacteria and Tenericutes have shown statistically significant SGD, irrespective of the trends in their nucleotide usages (Table 1). PolC are also present in Thermotogae members, but they do not possess PAS. In most cases, they have alternate R and Y-dominant stretches along their genome sequences (Trend III, Table 2). Our analysis shows that five out of seven Thermotogae species do not display any significant SGD. On the contrary, a large fraction of non-PolC organisms following Trend IV (i.e., G + T-dominance along LeS) have shown significant SGD – an observation that comply with earlier reports [16, 21]. These observations re-confirm that the presence of PolC is neither a necessary nor a sufficient condition for SGD in bacteria.

**Figure 6:**
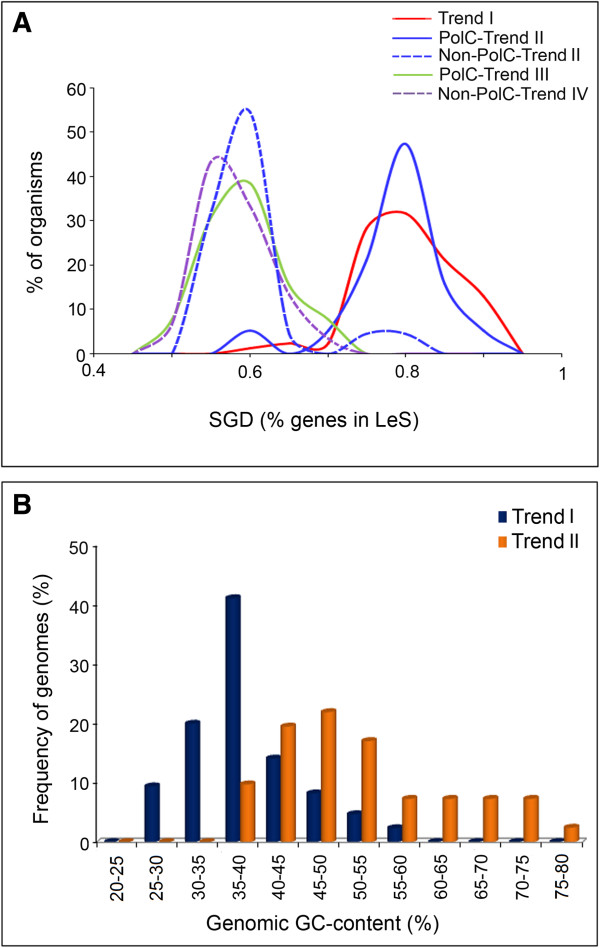
**SGD and Genomic GC-content distribution profiles in organisms showing different trends.** **(A)** Distribution of SGD in Trend I (red solid line), PolC-Trend II (blue solid line), non-PolC-Trend II (blue dotted line), PolC-Trend III (green solid line) and non-PolC Trend IV (violet dotted line) organisms; **(B)** Distribution of Genomic GC-content for Trend I and Trend II organisms.

The strength of SGD varies appreciably in organisms with different trends in nucleotide usages along their LeS/LaS, as can be seen from their SGD distribution profiles (Figure 6A) as well as from the individual SGD values (Table 1). Interestingly enough, the major peaks of the SGD distribution profiles of PolC-Trend I and PolC-Trend II organisms fall in the same range (~0.8) (Figure 6A), while the SGD profiles of the PolC-containing Trend III organisms, non-PolC Trend II organisms and non-PolC Trend IV organisms - all display peaks in the range of 0.55-0.6. In both Trend I and PolC-Trend II categories, SGD is greater than 0.7 for majority of the organisms in the dataset (Tables 1 and 2, Figure 6A). The only difference between two profiles is that in case of PolC-Trend II, there are a few genomes having SGD distribution profiles < 0.65, which could not be found in case of Trend I (Figure 6A). This observation indicates that organisms with only G-dominance may have relatively low SGD in some cases, but organisms showing explicit G + A-dominance are always characterized by a strong bias in gene orientation along replication direction. The strong resemblance between the distribution profile of Trend I organisms, characterized by PAS (and PolC) and that of PolC-Trend II organisms having G-dominance suggests that PAS asserts SGD, but SGD does not warrant PAS. For instance, the PolC-containing Thermoanaerobacterales species *Ammonifex degensii KC4* or Selenomonadales species *Veillonella parvula DSM 2008* do not show explicit PAS, but have extremely high SGD (>80% genes in LeS) (Table 1).The number of organisms in PolC-Trend III group is too low (one Firmicutes and twelve non-Firmicutes members) to provide any statistically significant pattern. Nevertheless, it is intriguing to find that the major peak of its SGD distribution profile comes in the same range as that of the non-PolC-Trend IV population. These distribution profiles give a hint that the average SGD of PolC-Trend III (and also of non-PolC-Trend II/non-PolC-Trend IV) organisms might not be as high as in cases of Trend I or PolC-Trend II (Figure 6A). In order to gain a conclusive picture on SGD profiles of PolC-Trend III genomes, one must wait for availability of complete genome sequence information for more number of species belonging to this category. Distribution profiles have not been plotted for PolC-Trend IV or Trend V organisms, since the current dataset contains only three organisms in Trend IV and four organisms in the Trend V categories.

### Distinct trends in base usage in three codon sites of Les and LaS genes and intergenic regions in Trend I and Trend II Firmicutes

**Figure 7:**
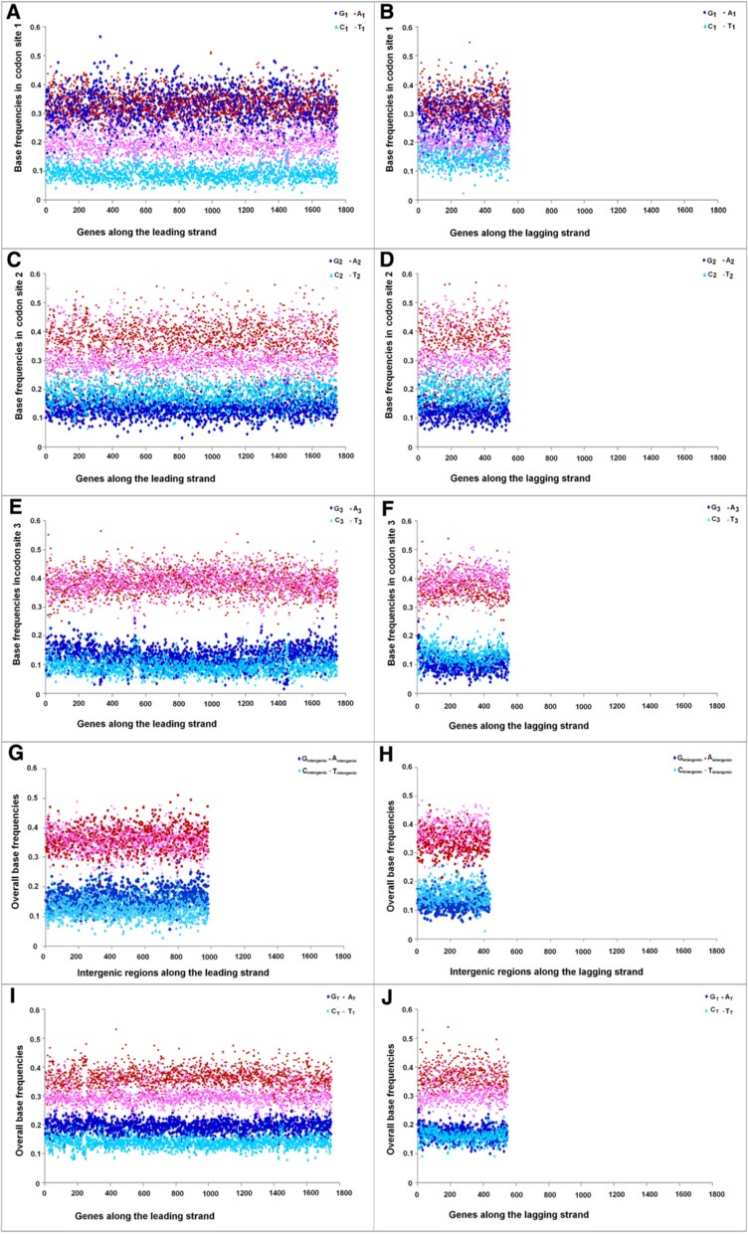
**Trends in individual base usages in** ***Staphylococcus aureus 04–02981*** **for genes encoded by both LeS and LaS.** Subscripts 1, 2, 3 indicate the percentage of occurrences of the respective base at 1st **(** **A** **,** **B** **)**, 2nd **(** **C** **,** **D** **)** and 3rd **(** **E** **,** **F** **)** codon sites, intergenic indicate the percentage of intergenic regions **(** **G** **,** **H** **)** and the subscript T stands for the total percentage **(** **I** **,** **J** **)** of occurrence of the base in individual genes of the organism.

**Figure 8:**
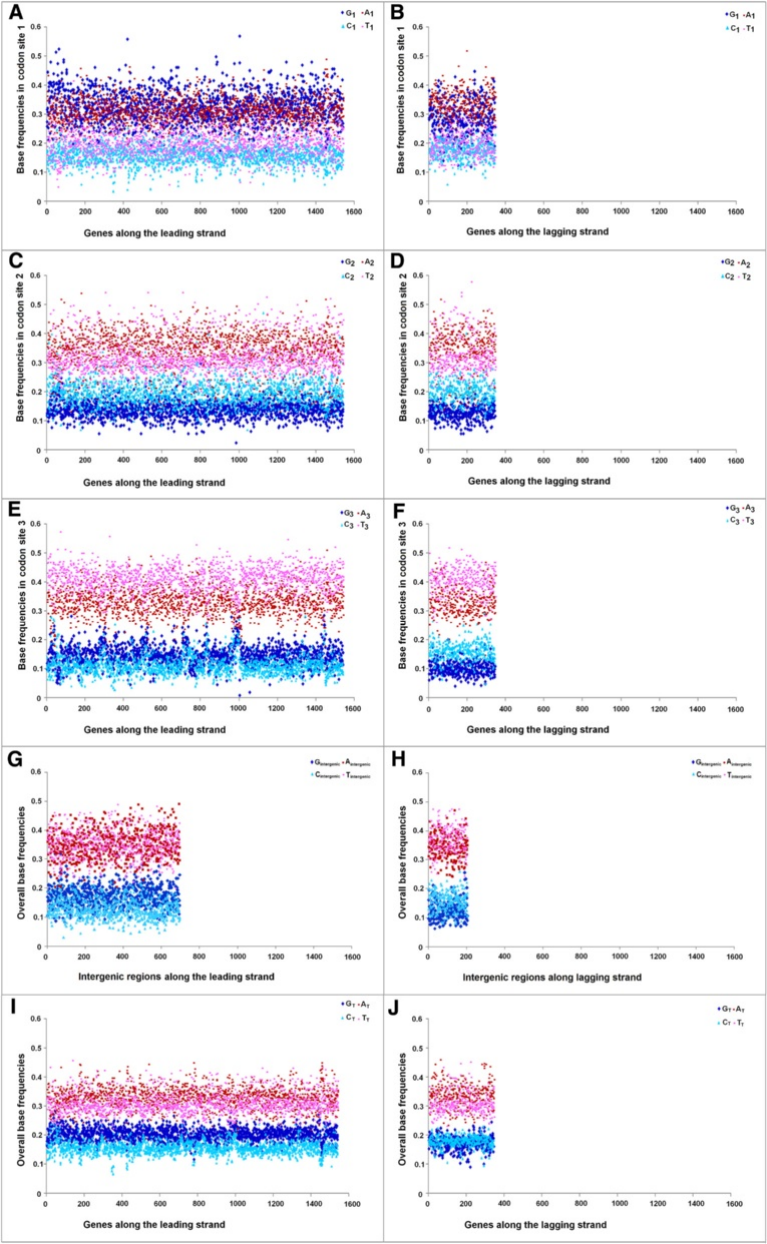
**Trends in individual base usages in** ***Streptococcus agalactiae NEM316*** **for genes encoded by both LeS and LaS.** Subscripts are same as in Figure 7.

**Figure 9:**
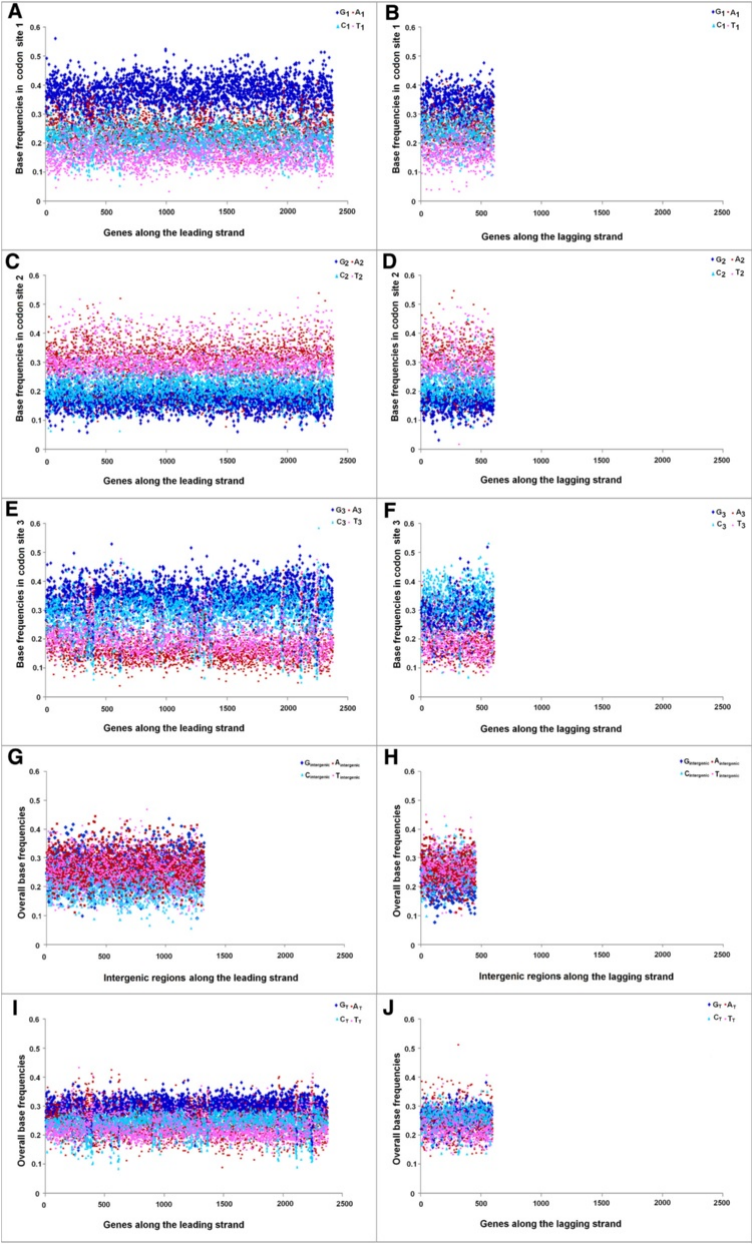
**Trends in individual base usages in** ***Geobacillus kaustophilus HTA426*** **for genes encoded by both LeS and LaS.** Subscripts are same as in Figure 7.

**Figure 10:**
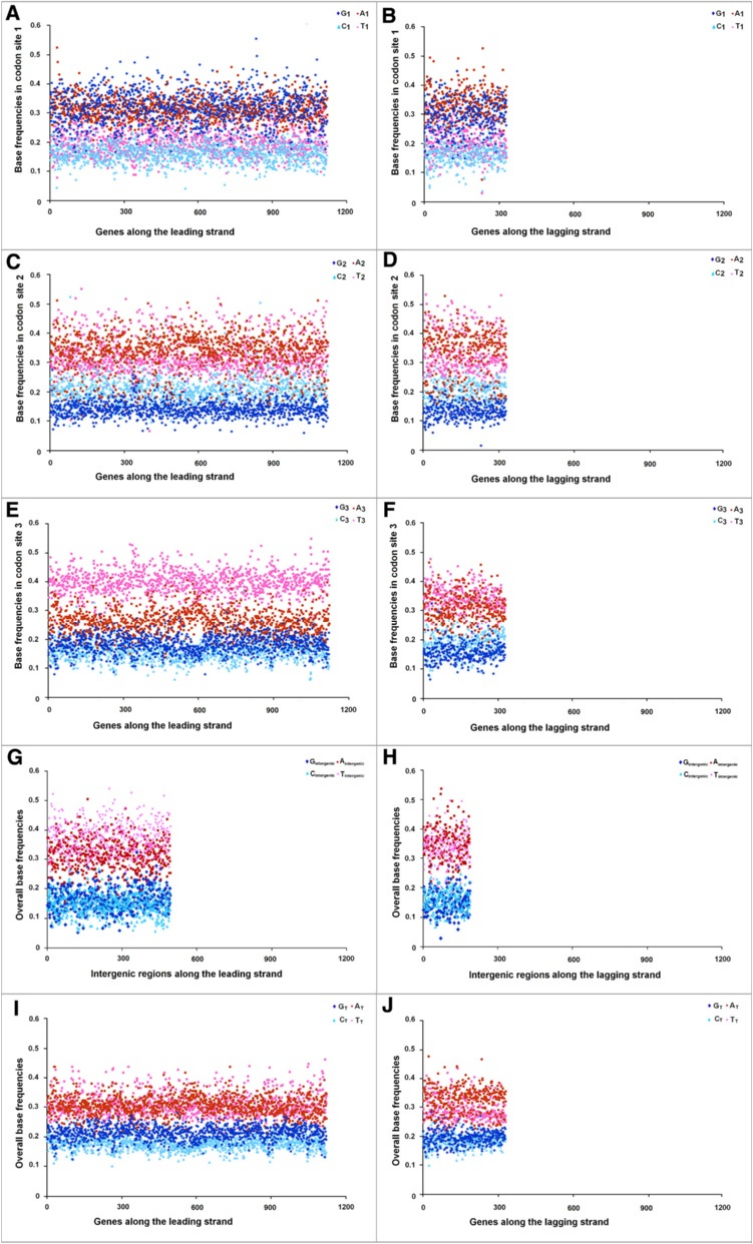
**Trends in individual base usages in** ***Oenococcus oeni PSU 1*** **for genes encoded by both LeS and LaS.** Subscripts are same as in Figure 7.

On the basis of strand-specific sequence composition, Firmicutes members may broadly be classified in two major categories: 1) the ones with G + A-dominance or PAS in LeS (Trend I) and 2) those having only G-dominance in LeS with no definite strand-specific bias in adenine usage (Trend II). There are some exceptions like *R. albus* or *O. onei* showing other conspicuous patterns in base usage (Trend III or Trend IV), but they are very few in number. Analysis of the distribution patterns of average genomic GC content of Trend I and Trend II organisms showed that the average GC-contents of Trend I organisms are usually significantly less than 50%, while the GC-content of Trend II genomes vary in much broader range (35 – 80%) (Figure 6B). It is not clear whether the relatively lower GC-content of the Trend I genomes could anyway be associated with PAS. This observation inspired us to further probe into the base usage patterns in three different codon sites of genes in two replicating strands of the Trend I and Trend II Firmicutes members of the current dataset. Figures 7, 8 and 9 represent three typical examples of the outcomes of this study. Figure 7 represents the trends in base usage in three individual codon sites and intergenic regions as well as in overall coding regions for all annotated genes in LeS (left panels) and LaS (right panels) of *S. aureus.* The organism is a typical representative of Trend I Firmicutes. Figures 8 and 9 depict the base usage patterns in *S. agalactiae* and *G. kaustrophilus* - two model representatives of Trend II Firmicutes with low and relatively high genomic GC-contents (35.6% and 52% respectively). Among the 102 Firmicutes species examined, only three species exhibited Trend IV. It is difficult to say whether the patterns observed in these three organisms typically represent the general trends in base usages within the PolC-containing Trend IV species of similar genomic G + C-content. Nevertheless, the base usage patterns in *O. onei* are shown in Figure 10 as a representative of these three species. The base usage in *E. coli* and *B. henselae* genes are depicted in Additional file 4: Figure S2 and Additional file 5: Figure S3 respectively, as the representatives of non-PolC organisms. *E. coli* represents Trend II non-PolC species, while *B. henselae* exemplifies Trend IV non-PolC organisms. There are usually no distinct strand-specific divergences in nucleotide usages in genes of Trend III or Trend V organisms (data not shown).

As revealed in Figures 7, 8, 9 and 10 and Additional file 4: Figure S2 and Additional file 5: Figure S3, there are some common features in base usages in organisms in general irrespective of their compositional trends. For instance, in most of the cases, G_1_ > C_1_ and A_1_ > T_1_, while G_2_ < C_2_ and A_2_ ≥ T_2_ in both LeS and LaS genes - an observation that conform with the existing notion of the universal three-base periodical pattern (G-non-G-N) of mRNA sequences [23]. Inter-group differences in base preferences are more apparent in the third codon sites of both LeS and LaS genes. There are some general patterns observed in 3rd codon sites of genes in PolC-containing organisms following Trend I - Trend III, as given below,

In Trend I species:

A_3_ ~ T_3_ > G_3_ > C_3_ (LeS genes), T_3_ ≥ A_3_ > C_3_ > G_3_ (LaS genes)

where, N_3_ indicates the average frequency of the nucleotide N in the 3rd codon sites of genes in the respective strands of the species under study.

In A + T-rich Trend II species:

T_3_ > A_3_ > G_3_ > C_3_ (LeS genes), T_3_ ≥ A_3_ > C_3_ > G_3_ (LaS genes)

In G + C-rich Trend II species:

G_3_ ≥ C_3_ > T_3_ > A_3_ (LeS genes), C_3_ ≥ G_3_ > T_3_ ~ A_3_ (LaS genes)

In *O. onei*, which represents the group of Trend IV organisms, especially of the A + T-rich ones:

T_3_ > A_3_ > G_3_ > C_3_ (LeS genes), T_3_ ≥ A_3_ > C_3_ ≥ G_3_ (LaS genes)

As shown in Additional file 4: Figure S2 and Additional file 5: Figure S3, trends in 3rd codon sites base usages in non-PolC organisms (both Trend II and Trend IV) are, by and large, similar to those observed in the PolC-containing Trend II organisms of similar G + C-bias, though the actual frequencies of different bases vary from one species to another.

In intergenic regions, usages of A and T are usually higher than those of G and C in most of the organisms (except in some highly G + C-rich organisms, where usages of A or T are comparable to usage of G or C). It was expected because of the presence of A + T-rich promoter sequences (TATA box etc.) in intergenic regions. Nevertheless, some specific biases in the base usages in the intergenic regions could be observed. For instance, in Trend I organisms, A_intergenic_ ~ T_intergenic_ along the LeS, but T_intergenic_ ≥ A_intergenic_ in LaS. This pattern is similar to that observed in the 3rd codon sites of the respective species. Furthermore, in most of the species, G_intergenic_ > C_intergenic_ along LeS, but C_intergenic_ > G_intergenic_ along LaS - a pattern observed in the 3rd codon sites the genic regions of the bacteria, in general, irrespective of their trends in base usages (Figures 7, 8, 9 and 10).

The overall base frequencies follow the trends, as given below.

In Trend I species,

A_T_ > T_T_ > G_T_ > C_T_ (LeS genes), A _T_ ≥ T_T_ > C_T_ ≥ G_T_ (LaS genes)

In A + T-rich Trend II species:

A_T_ ~ T_T_ > G_T_ > C_T_ (LeS genes), A_T_ ~ T_T_ > C _T_ > G_T_ (LaS genes)

In G + C-rich Trend II species,

G_T_ > C_T_ > A_T_ ~ T_T_ (LeS genes), C_T_ ≥ G_T_ > T_T_ ~ A_T_ (LaS genes)

In *O. onei* (Trend IV),

T_T_ ≥ A_T_ > G_T_ > C_T_ (LeS genes) A_T_ > T_T_ > G_T_ ~ C_T_ (LaS genes)These trends are in complete agreement with the GC-skew and AT-skew trajectories shown in Figures 1 and 2. Needless to say, a finite number of genes in each organism under study stand out as exceptions.

At a first glance, it may appear that base usage patterns in non-synonymous sites are quite similar across the two replicating strands of a particular species. However, a careful examination reveals some subtle differences. For instance, G_1_ in LeS genes is, in general, significantly higher than that in LaS genes of the same organism. On the contrary, C_1_ is, significantly lower in LeS genes as compared to that in LaS genes (in many cases, but not in all) (data not shown). Appreciable cross-strand differences in nucleotide selection have also been observed in the second codon sites of genes in a substantial number of PolC-containing organisms of the dataset (data not shown). The most prominent cross-strand difference in base usage is the preference for G over C by LeS genes and for C over G by LaS genes at their third codon sites (C_3_ ~ G_3_ in LaS genes in some cases, especially in GC-rich organisms).

## Discussion

The present study examines the status of PAS, SGD & PolC in Firmicutes and other bacterial species from diverse lineages. Co-existence of PAS, SGD and PolC in Firmicutes has earlier been reported by various investigators and several molecular mechanisms have been put forward as plausible explanations of this co-existence [1, 6, 10, 12]. Among these, the most accepted hypothesis is that the R-richness on the LeS and R-poorness on the LaS might be a type of sequence signature of the heterodimeric DNA polymerase III alpha subunit in Firmicutes [24]. It was also proposed that the presence of PolC might have exerted a selection pressure in favour of R-enrichment in LeS in order to prevent nonspecific RNA–RNA interactions and formation of excessive double-stranded RNA [22]. This, in turn, has led to the emergence of a strong SGD through preferential localization of R-rich genes in LeS during random genetic exchange across two strands [25]. On contrary to these existing notions, the present analysis clearly demonstrates that PAS or G + A-dominance in LeS is neither an essential feature of the Firmicutes, nor a sequence signature of PolC and/or SGD. It exists only in a subset of the Firmicutes, especially in those belong to the order *Bacillales*. There are an appreciable number of non-*Bacillales* Firmicutes (e.g., *Streptococcus*, *Geobacillus* or *Lactobacillus*), which contain PolC and have strong SGD.They do not show any definite strand-specific bias in their adenine usage patterns. In most of these Firmicutes, the cumulative R-content is significantly higher in the LeS than that in the LaS, but the sole contribution to R-asymmetry comes from the guanine bias, with little or no role of the adenine content. There is also a Firmicutes species *R. albus* that despite having PolC does not show strand-specific purine asymmetry. It rather contains alternate stretches of R-rich and Y-rich segments. Certain Firmicutes also exhibit G + T-dominance in their LeS sequences. It may therefore be said that PAS is not an essential feature of Firmicutes.

PAS is not an exclusive characteristic of the Firmicutes either. It has been observed in some Fusobacteria and Tenericutes species also. Among five Fusobacteria under study, three organisms namely *S. moniliformis, I. polytropus* and *S. termitidis*, exhibit strong PAS and strong SGD. The other two Fusobacteria members have alternate stretches of R*-*rich and R-poor regions along both the strands of replication, though all five members of the phylum possess PolC. Similarly, among twelve PolC-containing Tenericutes members of the dataset (Table 2), five species display strong PAS as well as highly significant SGD.

Observations made in the present study also suggest that the existence of PAS or G + A-richness of LeS is usually associated with PolC and a strong SGD, but the reverse may not be true. There are four bacterial phyla, namely Firmicutes, Fusobacteria, Tenericutes and Thermotogae, members of which contain PolC. Among these, PAS or G + A-richness of LeS prevails only in a certain fraction of Firmicutes and in three Fusobacteria, all of which carry PolC and almost all of which show strong SGD. However, there are a number of non-PAS Firmicutes, especially the ones exhibiting Trend II which also display equally strong SGD. It is therefore suggested that presence of a strong SGD does not necessarily imply PAS.

It was proposed earlier that PolC might play a role in maintenance of SGD in Firmicutes. The present study concords with this notion in the sense that majority of the PolC-containing genomes have significant SGD. However, the presence of PolC alone might not lead to a strong SGD (>70%). Most of the Trend III Firmicutes, Fusobacteria and Tenricutes members examined so far have shown relatively weak SGD (<70%). Interestingly enough, three Firmicutes species *B. tusiae*, *O. oeni, S, acidophilus,* having strong G + T dominance along their LeSs, exhibit the presence of strong SGD. It is, therefore, tempting to postulate that it might not be PolC alone, but a coupling between PolC and the G-dominance in LeS that has led to a strong SGD in the Firmicutes/Fusobacteria. Again, there are some exceptions. Two Clostridial species, *T. marianensis* and *D. acetoxidans* have SGD, but not PAS and PolC. It is intriguing to note that all Thermotogae members possess PolC and follow Trend III, but do not have any significant SGD. This observation indicates that the suggested correlation between PolC and SGD did not hold well in Thermotogae.

A comparison of the trends in base usages within different codon sites in PolC–containing Firmicutes (Figures 7, 8, 9 and 10) with those in non-PolC bacteria like *E. coli* (Additional file 4: Figure S2) or *B. henselae* (Additional file 5: Figure S3) reveals that the non-synonymous sites of genes follow certain general trends in most of these species; whereas the actual nucleotide frequencies vary from species to species depending on their average genomic GC-bias. However, a conspicuous trend that differentiates Trend I Firmicutes, Fusobacteria and Tenericutes from all other organisms; is similar or even higher usage of A_3_ as compared to that of T_3_ in LeS genes. It is in contrast to the earlier observations on preferences of pyrimidines over purines in third codon sites [26]. However, in all other organisms under study, usage of T_3_ is higher than that of A_3_ in LeS. These observations point to the existence of a unique selection pressure in Trend I Firmicutes in favour of adenine over thymine individually in all three codon sites, especially in the third ones. This unique feature of Trend I organisms seems to have a major contribution to the PAS.

Molecular processes that may incur strand-specific compositional biases in bacterial genomes include DNA replication, transcription coupled repair (TCR) [1, 3, 27–29] and the process of deamination and 5-methylation of cytosine [9, 21]. When a gene is located on the leading strand of a PolC-containing species, the mutational bias at the replication level and the bias at the transcription level both tend to increase its G + A-content; but the process of cytosine methylation generates a LeS-wide bias towards increasing G + T-content. On the contrary, genes on the LaS experience a mutational bias towards increasing C + A-content during the replicational process, a bias in favour of increasing G + A-content during TCR as well as a bias towards increasing C + T-content owing to the cytosine methylation. The resultant base composition of the LeS/LaS genes would depend on the relative intensities of these biases in the respective species. If all three processes remain significantly active in a genome, their collective effect is expected to create an unequivocal dominance of G over C in LeS genes of the organisms, as observed in Figures 7, 8, 9 and 10. If the mutational biases during replication and/or transcription dominate over the deamination/methylation bias, the frequencies of A would be higher than T. Thus it is tempting to propose that this might be the cases in Trend I organisms (Figure 7). On the other hand, if the G + T-bias owing to cytosine deamination be strong enough to nullify or even outshine the G + A-bias of replication/transcription processes, the LeS genes might exhibit Trend II or even Trend IV traits. Similar arguments may also be put forward to explain the compositional skews of LaS genes in Figures 7, 8, 9 and 10. Reports on the presence of a high level of α/β-type small, acid-soluble spore proteins (SASPs) in *Bacillus subtilis*[30] and in many other members of the orders Bacillales and Clostridiales [31, 32] suppressing cytosine deamination to uracil in native DNA are in good agreement with our proposition. Future investigations on the status and activities of the α/β-type SASPs in Trend II and Trend IV, which is out of the scope of the present analysis, may help in further validation of this notion.

In the entire dataset, there are only two Firmicutes members, which are devoid of two conspicuous features of the phyla, i.e., PAS and PolC. Considering the fact that bacterial genomes are highly dynamic in nature and they are continuously undergoing the processes of gene loss and gene gain, one could presume that the gene encoding PolC had been lost from these two Firmicutes members. Hence they did not experience any selection pressure in favour of PAS. Presence of SGD in these two organisms re-affirms that the existence of PAS or PolC is not an essential pre-requisite of SGD.

Among the non-Firmicutes, existence of PolC was reported earlier in *F. nucleatum* and *T. maritima* as potential cases of horizontal gene transfer [8, 33]. The present analysis indicates that PolC is present not only in these two species, but it is also shared with all other Fusobacteria and Thermotogae members examined in this study. In fact, among all non-Firmicutes in the current dataset, presence of PolC could so far be detected in three lineages – Fusobacteria, Mollicutes or Tenericutes and Thermotogae. Surprisingly enough, most the members of these three lineages exhibit strong explicit PAS (both G- and A-dominance in LeS) or have alternate R- and Y-dominance along their genomes (with a few exceptions that exhibit Trend V). It would not therefore be irrational to presume that the presence of PolC and the emergence of R-rich/Y-rich genome segments in some of these organisms might have some common link. It may be mentioned in this context that some of the earlier evolutionary studies pointed towards a plausible close evolutionary relationship among Firmicutes, Fusobacteria and Mollicutes. The ribosomal molecular phylogeny and core genome contents of Fusobacteria members indicated that this lineage might have branched out at the base of Firmicutes.

Mollicutes were previously thought to be a class within Firmicutes, but later on the basis of their unique phenotypic properties such as the lack of rigid cell walls and other evidences, they have been placed under a new phylum called Tenericutes [34]. However, the phylogenetic analysis based on phosphoglycerate kinase (Pgk) amino acid sequences indicated a monophyletic origin of the Mollicutes within Firmicutes [35]. The same study also had placed Fusobacteria (and even Thermotogae) within the Firmicutes – an observation that completely conforms to the findings made in the present study. One cannot, therefore, rule out the possibility that the feature of PAS was not horizontally acquired by the Fusobacteria or Mollicutes, but inherited normally from their Firmicutes like ancestors. Some members of Fusobacteria like *S. moniliformis, I. polytropus,* are still bearing the ancestral signature of PAS in their LeS sequences. However, their fellow members and the Mollicutes species might have undergone a series of genome reshuffling, recombination and local strand reversal processes in course of their evolution. As a consequence, their original ancestral genome architecture with R-rich LeS and R-poor LaS might have gradually been turned into the present-day genome structures having a mosaic of alternate *R*-rich and *R*-poor segments along both the strands. These processes of genome reshuffling or recombination might have also altered the gene orientation along two replicating strands. It would have been intriguing to study the correlations, if any between the processes of genome reshuffling and the evolution of gene orientation. However, it is beyond the scope of the present analysis.

The organisms showing Trend III or Trend V often exhibit zig-zag patterns in their GC-skew and other skew curves and it sometimes becomes difficult to identify the ter regions of their chromosomes unambiguously. One may argue that in such cases, a random pattern in base usages along two strands (Trend V) may arise due to an error in assignment of the ter region and hence among the LeS and LaS sequences. With a view to check whether it is mere shift in the ter region or mixing up of ancestral LeS and LaS sequences owing to genomic recombination that may alter the basic trend in base usage along LeS and LaS sequences, we have examined the GC-skew and AT-skew patterns (Additional file 6: Figure S4) in eight *Yerisina pestis* strains, which are known for having undergone drastic changes in the relative positions and directions of discrete genome segments following extensive genomic rearrangements [36]. In all strains except *Y. pestis Pestoides F*, putative oriC have been found near the start point of the reported plus strand sequences and the putative ter point, despite having finite displacement along plus strand, appeared to be located close to the mid-point of the plus strand. In *Y. pestis D182038* and *Y. pestis biovar Microtus 91001* yielding zig-zag cumulative GC-skew curves with multiple extrema, putative ter points were determined from the extremum point closest to the point representing the putative oriC plus half of the chromosome length (as described in the Methods section). *Y. pestis Pestoides F* is the only strain, where the putative oriC and ter regions (as detected from the unique extremum point of cumulative GC-skew) both have shifted in an uneven manner and as a consequence, the distances between oriC and ter points along two strands become significantly different (Additional file 6: Figure S4, HR). All the predicted locations of oriC and ter regions conform well to the findings made earlier by Liang et al. (Figure three of [36]). Interestingly enough, seven out of eight strains unambiguously exhibit Trend IV (Additional file 6: Figure S4, left panel, Table S3) and these include even *Y. pestis Pestoides F* having asymmetric locations of oriC and ter along the plus strand and *Y. pestis D182038* showing a zig-zag skew curve. The only exceptional case that displayed Trend V (Additional file 6: Table S3) is *Y. pestis biovar Microtus 91001* – the strain exhibiting maximum number of genomic rearrangement – translocation and/or inversion of 54 out of 61 genome plates with respect to the *Y. pestis CO92* genome, as reported in Figure 3 of Liang et al. [36]. This observation clearly indicated that it is neither an asymmetric location of oriC and ter regions, nor any ambiguity in the prediction of the ter point, but the specific types of genomic rearrangements leading to a substantial mixing up of LeS and LaS sequences that may result in a change in the trends in local base usages in bacterial genomes.

As already mentioned, the situation might have been quite different in case of Thermotogae. The exact position of Thermotogae within the tree of life is also not clear yet. Different markers have yielded varying results, which place Thermotogae and other hyperthermophiles like Aquificae either close to the root of the tree of life [37] or a little “up” from the root close to Fusobacteria [38] or to *Bacillus* and *Mycoplasma* species [39]. A significant degree of horizontal acquisition of genes by Thermotogae from other species, especially from archaea, has made the situation even more confusing. As already mentioned, the Pgk-based phylogeny, which was otherwise congruent with 16S rRNA data placed Thermotogales closer to Firmicutes than to any other phylum. In the light of all these studies, it may be said that there could be multiple events leading to the current architectures of Thermotogae genomes. PolC might have horizontally (or even vertically) acquired by an ancestral species prior to the branching of the lineage of Thermotogae and the current architecture of R-rich and R-poor segments of Thermotogae might be the relics of their ancestral PAS like sequence signature of the PolC. Alternately, considering the fact that Thermotogae are hyperthermophile in nature and that they are believed to be close enough to Aquificae, it is more likely that the presence of purine-rich and pyrimidine-rich stretches in Thermotogae rather reflects their molecular adaptation to high temperature.

## Conclusions

PAS, strong SGD and PolC should not be regarded as the signatures of the phylum of Firmicutes, as these features co-exist only in a subset of its members. Moreover, the features may occur, either collectively or individually in members of Fusobacteria, Tenericutes and Thermotogae as well. The study indicates that PAS might warrant the presence of PolC and strong SGD, but the presence of PolC or that of SGD not necessarily implies PAS. In other words, PAS might be a probable, but not an ordained outcome of PolC and strong SGD.

## Methods

### Sequence retrieval

All predicted protein coding sequences and the complete genome sequences of 102 Firmicutes members were retrieved from the NCBI GenBank. The organisms were chosen in a way to include representatives from all major subphyla and/or classes of the phylum of Firmicutes (Additional file 1: Table S1). Care had also been taken to keep the selection of organisms as varied as possible in terms of their characteristics lifestyle, habitat and genomic G + C-content. However, due to non uniform distribution of organisms of known genome sequences across different families of the Firmicutes, members from some family got overrepresented. Similarly 90 representative organisms of varying G + C-content and niche specificity from all other non-Firmicutes taxa (Additional file 2: Table S2) were also downloaded. All basic information of those organisms were collected from NCBI [40] and BacMap [41] databases.

For each organism under study, presumed duplicates, transposons and the annotated ORFs having less than 300 base pairs have been excluded from the dataset in order to reduce the stochastic errors,

### Segregation of two strands of replication (LeS and LaS) and evaluation of SGD in organisms under study

In order to segregate the LeS and LaS genes, one needs to determine the replication origin (oriC) or termination (ter) of the respective genome. It is well known that in bacteria, the base composition of each chromosomal strand changes at the origin and terminus of replication [13–15, 42–45], which is reflected in the change in sign in the cumulative GC-skew [(G-C)/G + C)] and other skew plots at oriC and ter [46–49]. With a view to determine oriC, the cumulative GC-skew analysis was performed with the help of an in-house developed program, using a sliding window of 10 Kb along the entire genome sequence of each species under examination. The oriC predicted from the extrema of the cumulative GC-skew were validated by checking the neighbouring gene organization along with the presence of DnaA boxes in their vicinity [46, 50], and also by comparing the same with the oriC sites of the respective genomes, as annotated in the DoriC database [51]. In most of the cases, the GenBank reference start point of the genome sequence turned out as the putative oriC, though there were a few exceptions.

The putative ter was then calculated as the location of the predicted oriC plus half of the length of the respective chromosome, as done previously by Mao et al. [52]. In majority of the organisms under study, the cumulative GC-skew changed the sign in the neighbourhood of the predicted ter, validating thereby the location of the ter region.

In some exceptional cases, especially in organisms following Trend III or Trend V, the cumulative GC-skew showed zig-zag trajectories with multiple extrema. The chromosomes of these organisms might have undergone large-scale genomic recombination, rearrangements and/or inversions, leading to a mixing of leading and lagging strands of replication and the zig-zag patterns of the cumulative GC-skew might be attributed to such genome rearrangement events. In such cases, the extremum point closest to the point representing the putative oriC plus half of the chromosome length was taken as the putative ter point. It may be argued that the oriC and ter sites in these organisms might undergo a shift from their original positions (i.e., prior to genetic rearrangements) and hence, the predicted oriC plus half of the chromosome length may not always represent the actual ter sites. However, shifting of ter sites would not change the general trends in base usage in such cases. A shift in oriC and/or ter would merely toggle the signs of local GC-skew and AT-skew. Since in Trend III organisms, most of the 10 kb windows have either both the skews positive or both negative and there would be no change in overall trend, if the skews toggle their signs simultaneously. On the other hand, the group of Trend V organisms includes all atypical cases of base combinations with no definite pattern and it is very unlikely that a shift in the oriC/ter sites would change an irregular pattern into a regular or well-defined one. This point has further been elaborated in the Discussion section, along with an example of *Yerisina pestis* strains, which have reportedly undergone substantial genetic rearrangements.

Based on the predicted oriC and ter sites, the two strands of replication were segregated by joining the oriC to ter region of one half of the plus strand with the ter to oriC region of the minus strand and vice-versa. The numbers of coding regions in two strands of replication were calculated for each genome and the strand with higher frequency of coding regions were taken as the LeS, following the usual convention [3, 52].

In order to ascertain SGD, a 2 × 2 chi-square contingency test was done with number of genes encoded by LeS and LaS, using STATISTICA (version 6.0, published by Statsoft Inc., Tulsa, Oklahoma, USA). Average G + C-content of each genome has also been calculated.

### Determination of instantaneous GC-skew, AT-skew and RY-skew for the sequenced genomes used in the study

The total purine-pyrimidine skew values [(R-Y)/(R + Y)] and instantaneous AT-skew values [(A-T)/(A + T)] were also calculated for a sliding window of 10 kb, using an in-house program and subsequent plots have been made.

Instantaneous GC-skew (blue color) and AT-skew (red color) values were plotted together against the respective windows along the genome sequence of each organism, in order to find out the distinct trends in purine/pyrimidine distributions. Some representatives of these plots are shown in Figures 1, 2, 3 and 4.The scatter plots of the instantaneous GC-skew and AT-skew values were also drawn in an attempt to affirm the nature of the trends in strand-specific purine and pyrimidine usages in LeS (blue color) and LaS (red color) of each genome, some representatives of which were shown in Figure 5.

### Classification of genomes according to the trends in base usage along the respective LeS and LaS sequences

With a view to classify the genomes under study according to the trends in base usage along their two strands of replication, the individual base frequencies were calculated for each sliding window of 10 kb along the LeS sequences. There could be four different combination of base usage in these LeS sequence segments as given below.

- frequency of G > frequency of C AND frequency of A > frequency of T.
- frequency of G > frequency of C AND frequency of A ≤ frequency of T.
- frequency of G ≤ frequency of C AND frequency of A > frequency of T.
- frequency of G ≤ frequency of C AND frequency of A ≤ frequency of T.

If there had been no strand-specific bias in base usage, the distribution of 10 kb LeS segments among these four possible combinations should have been uniform (around 25%), whatever be their average genomic GC-composition. But all genomes examined in the study showed distinct biases in distribution patterns of LeS segments among four groups. On the basis of observed biases in distribution of 10 kb LeS segments among above four groups, the organisms were classified into five distinct categories, as shown in Tables 1 and 2. The criteria for such classification are given below. Considering up to 5% deviations from the expected frequency of occurrence as normal stochastic variations, ‘random’ refers to frequencies in the normal range, i.e., (25 ± 5%), while ‘high’ and ‘low’ refer to frequencies >30% and <20% respectively.

- Trend I: (a) high, (b) random or low, (c) & (d) low → Enrichment of both G and A along LeS.
- Trend II: (a) & (b) high, (c) & (d) low → Only G-enrichment along LeS.
- Trend III: (a) high, (d) high or random, (b) & (c) low → Presence of both R-dominant & Y-dominant stretches along LeS.
- Trend IV: (b) high, (a) random or low, (c) & (d) low → G + T-richness of LeS.
- Trend V: all other possible cases such as (a)–(d) all random or (a) high, (b) & (d) random, or (b) high, (c) random etc. → No definite strand-specific bias.

Since these categorization criteria are based on the relative usages of G versus C and A versus T, they hold good for all types of genomes, irrespective of their average G + C-content.

### Determination of PolC orthologues in bacteria by BLASTP search

The annotation of PolC in all genomes under study was checked individually from their respective protein tables. There were three possibilities. In most of the PolC-containing species, the genes encoding PolC were unambiguously annotated and hence, could be taken as an evidence of presence of PolC in these organisms. In a few cases, products of some specific genes were marked as “putative DNA polymerase III alpha subunit” or “DNA polymerase III PolC-type”. In these cases, a BLASTP search was carried out with these particular gene sequences against a database of genomes belonging to the genus of the respective organism. Lastly, in cases where no PolC/ PolC-type/DNA Polymerase III alpha subunit gene or gene product could be found, we have taken the annotated PolC sequence(s) from other organisms (from closely related ones, wherever available) and a BLASTP search is carried against the whole genome sequence of the target organism. In both the cases, database hits with e value 0 to 10-e^20^, if any, were retained and considered as evidences of existence of PolC in the respective organisms.

### Determination of base usages at three codon positions and total sequences of individual genes and intergenic regions in leading and lagging strand of replication

Exhaustive base composition analysis was carried out to find out the individual base frequencies in three codon positions of each protein-coding regions of each genome under study, using the program CODONW 1.4.2 (written by John Peden and available at (http://sourceforge.net/projects/codonw/)). The individual purine (G + A) and pyrimidine (C + T) contents and the base frequencies for the total sequence of individual genes (G_T_, A_T_, C_T_, T_T_) were also calculated. The base usage patterns in intergenic regions (of length ≥ 100 bases) in LeS and LaS sequences of the genomes have also been determined. Since the intergenic regions flanked by the convergently or divergently transcribed genes cannot be unambiguously assigned to any specific strand of replication, only the non-coding sequences existing between two co-oriented genes (i.e., the flanking genes are either both transcribed from the leading strand or both from the lagging strand of replication) have been considered. Each of these base frequencies were then plotted against the respective orders of genes along LeS and LaS of the respective organisms (Figures 7, 8, 9 and 10, Additional file 4: Figure S2 and Additional file 5: Figure S3).Distribution curves of SGD and the histograms of the genomic G + C-contents (Figure 6A and B) were also plotted for different groups of organisms showing distinct trends in purine usage.

## Availability of supporting data

The data sets supporting the results of this article are included within the article (and its additional files).

## Electronic supplementary material

Additional file 1: Table S1: General features of Firmicutes used in this study.s (PDF 229 KB)

Additional file 2: Table S2: General features of non-Firmicutes used in this study. (PDF 89 KB)

Additional file 3: Figure S1: (L) Cumulative GC-skew (blue lines) and AT-skew (red lines) and (R) purine/pyrimidine skews (black lines) in some model representatives of Trend II organisms. (A) *Streptococcus agalactiae NEM316*, (B) *Acidaminococcus intestini RyC-MR95*, (C) *Geobacillus kaustophilus HTA426*, (D) *Veillonella parvula DSM 2008*, (E) *Thermodesulfobium narugense DSM 14796*, (F) *Clostridiales genomosp BVAB3 UPII9 5*, (G) *Acinetobacter sp.ADP1,* (H) *Candidatus Protochlamydia amoebophila UWE25*. (PDF 528 KB)

Additional file 4: Figure S2: Trends in individual base usages in *Escherichia coli str. K-12 substr. MG1655* for genes encoded by both LeS and LaS. Subscripts are same as in Figure 7. (PDF 688 KB)

Additional file 5: Figure S3: Trends in individual base usages in *Bartonella henselae str.Houston-1* for genes encoded by both leading and lagging strands*.* Subscripts are same as in Figure 7. (PDF 1 MB)

Additional file 6: Figure S4: (L) Instantaneous GC-skew (blue lines) and AT-skew (red lines) and (R) Cumulative GC-skew (blue lines) and AT-skew (red lines) in *Yerisina pestis* strains. (A) *Yersinia pestis CO92*, (B) *Yersinia pestis D106004*, (C) *Yersinia pestis D106004* (D) *Yersinia pestis Antiqua*, (E) *Yersinia pestis Nepal516*, (F) *Yersinia pestis KIM 10*, (G) *Yersinia pestis biovar Microtus 91001*, (H) *Yersinia pestis Pestoides F.* **Table S3**. Status of combinations (a) – (d) in *Y. pestis* strains under study. (PDF 1 MB)

## Abbreviations

Les: Leading strand
LaS: Lagging strand
PAS: Purine asymmetry
SGD: Strand-specific bias in gene distribution
TCR: Transcription-coupled repair.

## References

[1] Hu J, Zhao X, Yu J (2007). Replication-associated purine asymmetry may contribute to strand-biased gene distribution. Genomics.

[2] Qu H, Wu H, Zhang T, Zhang Z, Hu S, Yu J (2010). Nucleotide compositional asymmetry between the leading and lagging strands of eubacterial genomes. Res Microbiol.

[3] Rocha E (2002). Is there a role for replication fork asymmetry in the distribution of genes in bacterial genomes?. Trends Microbiol.

[4] Rocha EP, Danchin A (2003). Gene essentiality determines chromosome organisation in bacteria. Nucleic Acids Res.

[5] Rocha EP, Danchin A (2003). Essentiality, not expressiveness, drives gene-strand bias in bacteria. Nat Genet.

[6] Dervyn E, Suski C, Daniel R, Bruand C, Chapuis J, Errington J, Janniere L, Ehrlich SD (2001). Two essential DNA polymerases at the bacterial replication fork. Science.

[7] Bao Q, Tian Y, Li W, Xu Z, Xuan Z, Hu S, Dong W, Yang J, Chen Y, Xue Y, Xu Y, Lai X, Huang L, Dong X, Ma Y, Ling L, Tan H, Chen R, Wang J, Yu J, Yang H (2002). A complete sequence of the T. tengcongensis genome. Genome Res.

[8] Mira A, Pushker R, Legault BA, Moreira D, Rodriguez-Valera F (2004). Evolutionary relationships of Fusobacterium nucleatum based on phylogenetic analysis and comparative genomics. BMC Evol Biol.

[9] Lobry JR, Sueoka N (2002). Asymmetric directional mutation pressures in bacteria. Genome Biol.

[10] Charneski CA, Honti F, Bryant JM, Hurst LD, Feil EJ (2011). Atypical AT skew in Firmicute genomes results from selection and not from mutation. PLoS Genet.

[11] Paul S, Million-Weaver S, Chattopadhyay S, Sokurenko E, Merrikh H (2013). Accelerated gene evolution through replication-transcription conflicts. Nature.

[12] Wu H, Qu H, Wan N, Zhang Z, Hu S, Yu J (2012). Strand-biased gene distribution in bacteria is related to both horizontal gene transfer and strand-biased nucleotide composition. Genomics Proteomics Bioinformatics.

[13] Zhao X, Zhang Z, Yan J, Yu J (2007). GC content variability of eubacteria is governed by the pol III alpha subunit. Biochem Biophys Res Commun.

[14] Francino MP, Ochman H (1997). Strand asymmetries in DNA evolution. Trends Genet.

[15] Freeman JM, Plasterer TN, Smith TF, Mohr SC (1998). Patterns of genome organization in bacteria. Science.

[16] McLean MJ, Wolfe KH, Devine KM (1998). Base composition skews, replication orientation, and gene orientation in 12 prokaryote genomes. J Mol Evol.

[17] McInerney JO (1998). Replicational and transcriptional selection on codon usage in Borrelia burgdorferi. Proc Natl Acad Sci USA.

[18] Romero H, Zavala A, Musto H (2000). Codon usage in Chlamydia trachomatis is the result of strand-specific mutational biases and a complex pattern of selective forces. Nucleic Acids Res.

[19] Das S, Paul S, Chatterjee S, Dutta C (2005). Codon and amino acid usage in two major human pathogens of genus Bartonella–optimization between replicational-transcriptional selection, translational control and cost minimization. DNA Res.

[20] Das S, Paul S, Dutta C (2006). Evolutionary constraints on codon and amino acid usage in two strains of human pathogenic actinobacteria Tropheryma whipplei. J Mol Evol.

[21] Frank A, Lobry J (1999). Asymmetric substitution patterns: a review of possible underlying mutational or selective mechanisms. Gene.

[22] Lao PJ, Forsdyke DR (2000). Thermophilic bacteria strictly obey Szybalski’s transcription direction rule and politely purine-load RNAs with both adenine and guanine. Genome Res.

[23] Trifonov E (1987). Translation framing code and frame-monitoring mechanism as suggested by the analysis of mRNA and 16 S rRNA nucleotide sequences. J Mol Biol.

[24] Engelen S, Vallenet D, Medigue C, Danchin A (2012). Distinct co-evolution patterns of genes associated to DNA polymerase III DnaE and PolC. BMC Genomics.

[25] Bohlin J, Hardy S, Ussery D (2009). Stretches of alternating pyrimidine/purines and purines are respectively linked with pathogenicity and growth temperature in prokaryotes. BMC Genomics.

[26] Rapoport AE, Trifonov EN (2008). Excessive Clustering of Third Codon Position Pyrimidines in Prokaryotes. J Biomol Struct Dyn.

[27] Svejstrup JQ (2002). Mechanisms of transcription-coupled DNA repair. Nat Rev Mol Cell Biol.

[28] Baran RH, Ko H, Jernigan RW (2003). Methods for comparing sources of strand compositional asymmetry in microbial chromosomes. DNA Res.

[29] Necşulea A, Lobry JR (2007). A new method for assessing the effect of replication on DNA base composition asymmetry. Mol Biol Evol.

[30] Sohail A, Hayes CS, Divvela P, Setlow P, Bhagwat AS (2002). Protection of DNA by alpha/beta-type small, acid-soluble proteins from Bacillus subtilis spores against cytosine deamination. Biochemistry.

[31] Setlow P (2007). I will survive: DNA protection in bacterial spores. Trends Microbiol.

[32] Paredes-Sabja D, Setlow P, Sarker MR (2011). Germination of spores of Bacillales and Clostridiales species: mechanisms and proteins involved. Trends Microbiol.

[33] Nelson KE, Clayton RA, Gill SR, Gwinn ML, Dodson RJ, Haft DH, Hickey EK, Peterson JD, Nelson WC, Ketchum KA (1999). Evidence for lateral gene transfer between Archaea and bacteria from genome sequence of Thermotoga maritima. Nature.

[34] Ludwig W, Schleifer K-H, Whitman WB (2009). Revised road map to the phylum Firmicutes. Bergey’s Manual® of Systematic Bacteriology.

[35] Wolf M, Muller T, Dandekar T, Pollack JD (2004). Phylogeny of Firmicutes with special reference to Mycoplasma (Mollicutes) as inferred from phosphoglycerate kinase amino acid sequence data. Int J Syst Evol Microbiol.

[36] Liang Y, Hou X, Wang Y, Cui Z, Zhang Z, Zhu X, Xia L, Shen X, Cai H, Wang J (2010). Genome rearrangements of completely sequenced strains of Yersinia pestis. J Clin Microbiol.

[37] Brown JR, Douady CJ, Italia MJ, Marshall WE, Stanhope MJ (2001). Universal trees based on large combined protein sequence data sets. Nat Genet.

[38] Brochier C, Philippe H (2002). Phylogeny: a non-hyperthermophilic ancestor for bacteria. Nature.

[39] Daubin V, Gouy M, Perriere G (2002). A phylogenomic approach to bacterial phylogeny: evidence of a core of genes sharing a common history. Genome Res.

[40] Pruitt KD, Tatusova T, Maglott DR (2007). NCBI reference sequences (RefSeq): a curated non-redundant sequence database of genomes, transcripts and proteins. Nucleic Acids Res.

[41] Stothard P, Van Domselaar G, Shrivastava S, Guo A, O’Neill B, Cruz J, Ellison M, Wishart DS (2005). BacMap: an interactive picture atlas of annotated bacterial genomes. Nucleic Acids Res.

[42] Arakawa K, Suzuki H, Tomita M (2009). Quantitative analysis of replication-related mutation and selection pressures in bacterial chromosomes and plasmids using generalised GC-skew index. BMC Genomics.

[43] Grigoriev A (1998). Analyzing genomes with cumulative skew diagrams. Nucleic Acids Res.

[44] Lobry JR (1996). Asymmetric substitution patterns in the two DNA strands of bacteria. Mol Biol Evol.

[45] Lobry J (1996). A simple vectorial representation of DNA sequences for the detection of replication origins in bacteria. Biochimie.

[46] Sernova NV, Gelfand MS (2008). Identification of replication origins in prokaryotic genomes. Brief Bioinform.

[47] Song J, Ware A, Liu S-L (2003). Wavelet to predict bacterial ori and ter: a tendency towards a physical balance. BMC Genomics.

[48] Zhang R, Zhang C-T (2005). Identification of replication origins in archaeal genomes based on the Z-curve method. Archaea.

[49] Grigoriev A (2000). Graphical genome comparison: rearrangements and replication origin of Helicobacter pylori. Trends Genet.

[50] Mackiewicz P, Zakrzewska-Czerwinska J, Zawilak A, Dudek MR, Cebrat S (2004). Where does bacterial replication start? Rules for predicting the oriC region. Nucleic Acids Res.

[51] Gao F, Luo H, Zhang C-T (2013). DoriC 5.0: an updated database of oriC regions in both bacterial and archaeal genomes. Nucleic Acids Res.

[52] Mao X, Zhang H, Yin Y, Xu Y (2012). The percentage of bacterial genes on leading versus lagging strands is influenced by multiple balancing forces. Nucleic Acids Res.

