# Supplementary material for: Association of purine asymmetry, strand-biased gene distribution and PolC within Firmicutes and beyond: a new appraisal
Source: BMC Genomics. 2014 Jun 4;15(1):430. doi: 10.1186/1471-2164-15-430 (PMC4070872; doi:10.1186/1471-2164-15-430)
Supplement: Supplementary file 1 — Additional file 1: Table S1: General features of Firmicutes used in this study.s (PDF 229 KB) [file 12864_2013_6136_MOESM1_ESM.pdf]

Additional data file 1: Table S1. General features of Firmicutes used in this study.

Class	Order	Family	Organism	Accession No. (Ref_Seq)	Genome Size (MB)	GC-content (%)	Temperature range	Habitat	Oxygen requirement
BA	BAC	ALIC	*Alicyclobacillus acidocaldarius subsp. acidocaldarius DSM 446*	NC_013205.1	3.21	61.9	Thermophilic	Specialized	Aerobic
			Bacillus tusciae DSM 2912	NC_014098.1	3.38	59.1	Thermophilic	*	Aerobic
		BACI	*Anoxybacillus flavithermus WK1*	NC_011567.1	2.85	41.8	Thermophilic	Specialized	Facultative
			Bacillus amyloliquefaciens FZB42	NC_009725.1	3.9	46.5	Mesophilic	Terrestrial	Aerobic
			Bacillus anthracis str. Ames	NC_003997.3	5.2	35.4	Mesophilic	Multiple	Facultative
			Bacillus atrophaeus 1942	NC_014639.1	4.17	43.2	Mesophilic	Soil	Aerobic
			Bacillus cellulosilyticus DSM 2522	NC_014829.1	4.68	36.5	Mesophilic	Multiple	Aerobic
			Bacillus cereus ATCC 10987	NC_003909.1	5.41	35.5	Mesophilic	Multiple	Aerobic
			Bacillus clausii KSM-K16	NC_006582.1	4.3	44.8	Mesophilic	Soil	*
			Bacillus cytotoxicus NVH 391-98	NC_009674.1	4.09	35.9	Mesophilic	Terrestrial	Aerobic
			Bacillus halodurans C-125	NC_002570.2	4.20	43.7	Mesophilic	Multiple	Facultative
			Bacillus licheniformis ATCC 14580	NC_006270.3	4.20	46.2	Mesophilic	Terrestrial	Facultative
			Bacillus megaterium DSM319	NC_014103.1	5.10	38.1	Mesophilic	Multiple	Aerobic
			Bacillus pseudofirmus OF4	NC_013791.1	4.25	39.9	Mesophilic	Terrestrial	Facultative
			Bacillus pumilus SAFR-032	NC_009848.1	3.7	41.3	Mesophilic	Terrestrial	Aerobic
			Bacillus selenitireducens MLS10	NC_014219.1	3.59	48.7	Mesophilic	Multiple*	Facultative
			Bacillus subtilis subsp. subtilis str. 168	NC_000964.2	4.22	43.5	Mesophilic	Terrestrial	Facultative
			Bacillus thuringiensis serovar konkukian str. 97-27	NC_005957.1	5.31	35.4	Mesophilic	Multiple	Facultative
			Bacillus weihenstephanensis KBAB4	NC_010184.1	5.87	35.5	Psychrophilic	Soil	Facultative
			Geobacillus kaustophilus HTA426	NC_006510.1	3.59	52.0	Thermophilic	Aquatic	Aerobic
			Halobacillus halophilus DSM 2266	NC_017668.1	4.17	41.8	Mesophilic	Soil	Anaerobic
			Lysinibacillus sphaericus C3-41	NC_010382.1	4.82	37.1	Mesophilic	Specialized	Aerobic
			Oceanobacillus iheyensis HTE831	NC_004193.1	3.63	35.7	Mesophilic	Multiple	Aerobic
		BACF	*Exiguobacterium AT1b*	NC_012673.1	3.00	48.5	Thermophilic	Specialized	Facultative
			Exiguobacterium sibiricum 255 15	NC_010556.1	3.04	47.7	Psychrophilic	Specialized	Facultative
		LIST	*Listeria innocua Clip11262*	NC_003212.1	3.09	37.3	Mesophilic	Soil	Facultative
			Listeria monocytogenes 07PF0776	NC_017728.1	2.90	38.0	Mesophilic	Multiple	Facultative
			Listeria seeligeri serovar 1/2b str. SLCC3954	NC_013891.1	2.80	37.4	Mesophilic	Soil	Facultative
		OCEA	*Oenococcus oeni PSU 1*	NC_008528.1	1.78	37.9	Mesophilic	Multiple	Facultative
		PAEN	*Brevibacillus brevis NBRC 100599*	NC_012491.1	6.30	47.3	Mesophilic	Terrestrial	Aerobic
			Paenibacillus polymyxa E681	NC_014483.1	5.39	45.8	Mesophilic	Terrestrial	Aerobic
			Paenibacillus Y412MC10	NC_013406.1	7.12	51.2	Mesophilic	Host associated	Aerobic
		PLAN	*Solibacillus silvestris StLB046*	NC_018065.1	3.98	38.6	Mesophilic	*	Aerobic
		STAP	*Staphylococcus aureus 04-02981*	NC_017340.1	2.82	32.9	Mesophilic	Host associated	Facultative
			Staphylococcus epidermidis ATCC 12228	NC_004461.1	2.56	32.1	Mesophilic	Host associated	Facultative
			Staphylococcus haemolyticus JCSC1435	NC_007168.1	2.70	32.8	Mesophilic	Host associated	Facultative
			Staphylococcus lugdunensis HKU09-01	NC_013893.1	2.66	33.9	Mesophilic	Host associated	Facultative
	LAC	AERO	*Aerococcus urinae ACS-120-V-Col10a*	NC_015278.1	2.08	41.9	Mesophilic	Host associated	Anaerobic
		CARN	*Carnobacterium sp. 17-4*	NC_015391.1	2.69	35.2	Mesophilic	Marine	*
		ENTE	*Enterococcus faecalis V583*	NC_004668.1	3.36	37.4	Mesophilic	Multiple	Facultative
			Enterococcus faecium DO	NC_017960.1	3.05	37.9	Mesophilic	Multiple	Facultative
		LACT	*Lactobacillus acidophilus NCFM*	NC_006814.3	1.99	34.7	Mesophilic	Host associated	Facultative
			Lactobacillus amylovorus GRL 1112	NC_014724.1	2.13	38.1	Mesophilic	Host associated	Facultative
			Lactobacillus brevis ATCC 367	NC_008497.1	2.34	46.0	Mesophilic	Host associated	Facultative
			Lactobacillus gasseri ATCC 33323	NC_008530.1	1.89	35.3	Mesophilic	Host associated	Facultative
		LEUC	*Leuconostoc kimchii IMSNU 11154*	NC_014136.1	2.10	37.9	Psychrotrophic	*	Facultative
			Leuconostoc mesenteroides subsp. mesenteroides ATCC 8293	NC_008531.1	2.08	37.7	Mesophilic	Multiple	Facultative
		STRE	*Lactococcus garvieae Lg2*	NC_017490.1	1.96	38.8	Mesophilic	Host associated	Facultative
			Lactococcus lactis subsp. cremoris MG1363	NC_009004.1	2.53	35.7	Mesophilic	Multiple	Facultative
			Streptococcus agalactiae NEM 316	NC_004368.1	2.21	35.6	Mesophilic	Host associated	Facultative
			Streptococcus equi subsp. zooepidemicus	NC_012470.1	2.50	41.5	Mesophilic	Host associated	Facultative
			Streptococcus pneumoniae D39	NC_008533.1	2.05	39.7	Mesophilic	Host associated	Facultative
			Streptococcus pyogenes MGAS10394	NC_006086.1	1.90	38.7	Mesophilic	Host associated	Facultative
CL	CLO	CLOS	*Alkaliphilus metalliredigens QYMF*	NC_009633.1	4.93	36.8	Mesophilic	Ponds	Anaerobic
			Clostridium acetobutylicum ATCC 824	NC_003030.1	4.13	30.9	Mesophilic	Soil	Obligate anaerobic
			Clostridium autoethanogenum DSM 10061	NC_022592.1	4.35	31.1	*	*	*
			Clostridium beijerinckii NCIMB 8052	NC_006177.1	6.00	29.9	Mesophilic	Multiple	Anaerobic
			Clostridium botulinum A ATCC 19397	NC_009697.1	3.86	28.2	Mesophilic	Multiple	Obligate anaerobic
			Clostridium novyi NT	NC_008593.1	2.55	28.9	Mesophilic	Terrestrial	Anaerobic
		CLOF	*Anaerococcus prevotii DSM 20548*	NC_013171.1	2.00	35.7	Mesophilic	Host associated	Anaerobic
			Finegoldia magna ATCC 29328	NC_010376.1	1.99	32.1	Mesophilic	Host associated	Anaerobic
			Sulfobacillus acidophilus DSM 10332	NC_016884.1	3.56	56.8	Thermophilic	Soil	Anaerobic
			Symbiobacterium thermophilum IAM 14863	NC_006177.1	3.57	68.7	Thermophilic	Soil	Anaerobic
			Thermaerobacter marianensis DSM 12885	NC_014831.1	2.84	72.5	Hyperthermophilic	Mud	Obligate aerobic
		EUBA	*Acetobacterium woodii DSM 1030*	NC_016894.1	4.04	39.3	Mesophilic	Specialized	Anaerobic
			Eubacterium rectale ATCC 33656	NC_012781.1	3.45	41.5	Mesophilic	Host associated	Anaerobic

		LACH	*Butyrivibrio proteoclasticus B316*	NC_014387.1	4.40	40.0	Mesophilic	Host associated	Anaerobic
			Clostridium lentocellum DSM 5427	NC_015275.1	4.71	34.3	Mesophilic	Aquatic	Anaerobic
			Roseburia hominis A2-183	NC_015977.1	3.59	48.5	Mesophilic	Host associated	Anaerobic
		OSCI	*Oscillibacter valericigenes Sjm18-20*	NC_016048.1	4.47	53.2	Mesophilic	Host associated	Anaerobic
		PEPT	*Desulfitobacterium hafniense DCB 2*	NC_011830.1	5.28	47.5	Mesophilic	Host associated	Anaerobic
			Desulfotomaculum acetoxidans DSM 771	NC_013216.1	4.55	41.6	Mesophilic	Multiple	Obligate anaerobic
			Desulfotomaculum reducens MI 1	NC_009253.1	3.61	42.3	Mesophilic	Aquatic	Anaerobic
			Desulfotomaculum ruminis DSM 2154	NC_015589.1	3.97	47.2	Mesophilic	Multiple	Anaerobic
		PEPP	*Clostridium cellulovorans 743B*	NC_014393.1	5.26	31.2	Mesophilic	Soil	Anaerobic
			Clostridium difficile CD196	NC_013315.1	4.11	28.6	Mesophilic	Host associated	Obligate anaerobic
			Clostridium sticklandii DSM 519	NC_014614.1	2.72	33.3	Mesophilic	Terrestrial	Anaerobic
		RUMI	*Ethanoligenens harbinense YUAN-3*	NC_014828.1	3.01	55.6	Mesophilic	Sludge	Obligate anaerobic
			Ruminococcus albus 7	NC_014833.1	4.48	43.6	Mesophilic	Host associated	Anaerobic
		SYNT	*Syntrophomonas wolfei subsp. wolfei str. Goettingen*	NC_008346.1	2.94	44.9	Mesophilic	Sludge	Anaerobic
			Syntrophothermus lipocalidus DSM 12680	NC_014220.1	2.41	51.0	Thermophilic	Waste water sludge	Obligate anaerobe
		UCLO	*Clostridiales genomosp BVAB3 UPII9 5*	NC_013895.2	1.81	44.2	*	Host associated	*
	THE	HALA	*Acetohalobium arabaticum DSM 5501*	NC_014378.1	2.47	36.6	Mesophilic	Aquatic	Anaerobic
			Halanaerobium hydrogeniformans	NC_014654.1	2.61	33.2	Mesophilic	Aquatic	Obligate anaerobic
		NATR	*Natranaerobius thermophilus JW/NM-WN-LF*	NC_010718.1	3.19	36.3	Thermophilic	Aquatic	Obligate anaerobic
		THER	*Ammonifex degensii KC4*	NC_013385.1	2.16	59.4	Thermophilic	Specialized	Anaerobic
			Caldicellulosiruptor bescii DSM 6725	NC_012034.1	2.93	35.2	Thermophilic	Specialized	Obligate anaerobic
			Caldicellulosiruptor hydrothermalis 108	NC_014652.1	2.77	36.1	Hyperthermophilic	Specialized	Anaerobic
			Carboxydothermus hydrogenoformans Z-2901	NC_007503.1	2.40	42.0	Hyperthermophilic	Specialized	Anaerobic
			Moorella thermoacetica ATCC 39073	NC_007644.1	2.63	55.8	Thermophilic	Aquatic	Anaerobic
			Tepidanaerobacter acetatoxydans Re1	NC_015519.1	2.76	37.3	Mesophilic	Sludge	Anaerobic
			Thermoanaerobacter pseudethanolicus ATCC 33223	NC_010321.1	2.36	34.5	Thermophilic	Aquatic	Anaerobic
			Thermoanaerobacter tengcongensis MB4	NC_003869.1	2.69	37.6	Hyperthermophilic	Specialized	Anaerobic
		THEF	*Caldicellulosiruptor saccharolyticus DSM 8903*	NC_009437.1	2.97	35.3	Hyperthermophilic	Specialized	Anaerobic
		THED	*Coprothermobacter proteolyticus DSM 5265*	NC_011295.1	1.42	44.8	Thermophilic	Specialized	Anaerobic
			Thermodesulfobium narugense DSM 14796	NC_015499.1	1.90	33.9	Thermophilic	Hot spring	Anaerobic
ER	ERY	ERYS	*Erysipelothrix rhusiopathiae str. Fujisawa*	NC_015601.1	1.79	36.6	Mesophilic	Host associated	Facultative
NE	SEL	ACID	*Acidaminococcus fermentans DSM 20731*	NC_013740.1	2.33	55.8	Mesophilic	Multiple	Anaerobic
			Acidaminococcus intestini RyC-MR95	NC_016077.1	2.49	50.0	Mesophilic	Host associated	Anaerobic
		VEIL	*Selenomonas ruminantium subsp. lactilytica TAM6421*	NC_017068.1	3.63	50.2	Mesophilic	Host associated	Anaerobic
			Selenomonas sputigena ATCC 35185	NC_015437.1	2.57	57.1	Mesophilic	Host associated	Anaerobic
			Veillonella parvula DSM 2008	NC_013520.1	2.13	38.6	Mesophilic	Host associated	Anaerobic

BA - Bacilli, CL - Clostridia, ER - Erysipelotrichi, NE - Negativicutes, BAC - Bacillales, LAC - Lactobacillales, CLO - Clostridiales, THE - Thermoanaerobacterales, ERY - Erysipelotrichales, SEL – Selenomonadales, ALIC - Alicyclobacillaceae, BACI - Bacillaceae, BACF - Bacillales Family XII. Incertae Sedis, LIST – Listeriaceae, OCEA – Oceanobacillus, PAEN – Paenibacillaceae, PLAN – Planococcaceae, STAP – Staphylococcus, AERO – Aerococcaceae, CARN – Carnobacteriaceae, ENTE – Enterococcaceae, LACT – Lactobacillaceae, LEUC – Leuconostocaceae, STRE – Streptococcaceae, CLOS – Clostridiaceae, CLOF - Clostridiales Family XI. Incertae Sedis, EUBA – Eubacteriaceae, LACH – Lachnospiraceae, OSCI – Oscillospiraceae, PEPT – Peptococcaceae, PEPP – Peptostreptococcaceae, RUMI – Ruminococcaceae, SYNT – Syntrophomonadaceae, UCLO - Unclassified Clostridiales, HALA – Halanaerobiaceae, NATR – Natranaerobiales, THER – Thermoanaerobacteraceae, THEF - Thermoanaerobacterales Family III. Incertae Sedis, THED – Thermodesulfobiaceae, ERYS – Erysipelotrichaceae, ACID – Acidaminococcaceae, VEIL – Veillonellacea.

* - Information not available.
