# Supplementary material for: Association of purine asymmetry, strand-biased gene distribution and PolC within Firmicutes and beyond: a new appraisal
Source: BMC Genomics. 2014 Jun 4;15(1):430. doi: 10.1186/1471-2164-15-430 (PMC4070872; doi:10.1186/1471-2164-15-430)
Supplement: Supplementary file 2 — Additional file 2: Table S2: General features of non-Firmicutes used in this study. (PDF 89 KB) [file 12864_2013_6136_MOESM2_ESM.pdf]

Additional data file 2: Table S2. General features of non-Firmicutes used in this study.

Organism	Taxonomy	Accession No. (Ref_Seq)	Genome Size (MB)	GC - content (%)	Temperature range	Habitat	Oxygen requirements
Acidobacterium capsulatum ATCC 51196	Acidobacteria	NC_012483.1	4.13	60.5	Mesophilic	Multiple	Aerobic
Candidatus Solibacter usitatus Ellin6076		NC_008536.1	9.97	61.9	Mesophilic	Terrestrial	Aerobic
Bifidobacterium longum NCC2705	Actinobacteria	NC_004307.2	2.26	60.1	Mesophilic	Host associated	Anaerobic
Leifsonia xyli CTCB07		NC_006087.1	2.58	67.7	Mesophilic	Host associated	Aerobic
Mycobacterium tuberculosis CDC1551		NC_002755.2	4.40	65.6	Mesophilic	Host associated	Aerobic
Nocardia farcinica IFM 10152		NC_006361.1	6.29	70.7	Mesophilic	Multiple	Aerobic
Streptomyces coelicolor A3 2		NC_003888.3	9.05	72.0	Mesophilic	Multiple	Aerobic
Anaplasma phagocytophilum HZ	Alphaproteobacteria	NC_007797.1	1.47	41.6	Mesophilic	Host associated	Aerobic
Neorickettsia sennetsu str. Miyayama		NC_007798.1	0.86	41.1	Mesophilic	Host associated	Aerobic
Wolbachia endosymbiont of Drosophila melanogaster		NC_002978.6	1.27	35.2	Mesophilic	Host associated	Aerobic
Zymomonas mobilis subsp. mobilis NCIMB 11163		NC_013355.1	2.20	46.6	Mesophilic	*	Facultative
Bartonella henselae str. Houston-1		NC_005956.1	1.93	38.2	Mesophilic	Host associated	Aerobic
Sinorhizobium meliloti 1021		NC_003047.1	6.70	62.2	Mesophilic	Multiple	Aerobic
Hydrogenobaculum Y04AAS1	Aquificae	NC_011126.1	1.56	34.8	Thermophilic	Aquatic	Aerobic
Persephonella marina EX-H1		NC_012440.1	1.98	37.1	Thermophilic	Multiple	Microaerophilic
Sulfurihydrogenibium YO3AOP1		NC_010730.1	1.84	32.0	Thermophilic	Specialized	Facultative
Aquifex aeolicus VF5		NC_000918.1	1.59	43.3	Hyperthermophilic	Specialized	Aerobic
bacteroides fragilis 638R	Bacteroidetes/Chlorobi	NC_016776.1	5.40	43.4	Mesophilic	Host associated	Obligate anaerobic
Chlorobium tepidum TLS		NC_002932.3	2.15	56.5	Thermophilic	Specialized	Obligate anaerobic
Croceibacter atlanticus HTCC2559		NC_014230.1	3.00	33.9	Mesophilic	Aquatic	Aerobic
Porphyromonas gingivalis W83		NC_002950.2	2.34	48.3	Mesophilic	Host associated	Anaerobic
Salinibacter ruber DSM 13855		NC_007677.1	3.59	66.1	Mesophilic	Specialized	Aerobic
Aromatoleum aromaticum EbN1	Betaproteobacteria	NC_006513.1	4.73	64.7	Mesophilic	Multiple	Facultative
Bordetella bronchiseptica RB50		NC_002927.3	5.34	68.1	Mesophilic	Host associated	Obligate anaerobic
Neisseria meningitidis MC58		NC_003112.2	2.27	51.5	Mesophilic	Host associated	Aerobic
Nitrosomonas europaea ATCC 19718		NC_004757.1	2.81	50.7	Mesophilic	Multiple	Aerobic
Polynucleobacter necessarius subsp. asymbioticus QLW-P1DMWA-1		NC_009379.1	2.16	44.8	Mesophilic	Multiple	Aerobic
Ralstonia solanacearum GMI1000		NC_003295.1	5.80	67.0	Mesophilic	Multiple	Aerobic
Candidatus Protochlamydia amoebophila UWE25	Chlamydiae/Verrucomicrobia	NC_005861.1	2.41	34.7	Mesophilic	Host associated	*
Chlamydia trachomatis A/HAR-13		NC_007429.1	1.05	41.3	Mesophilic	Host associated	*
Chlamydophila caviae GPIC		NC_003361.3	1.18	39.2	Mesophilic	Host associated	*
Waddlia chondrophila WSU 86 1044		NC_014225.1	2.13	43.8	*	Host associated	*
Chloroflexus aggregans DSM 9485	Chloroflexi	NC_011831.1	4.70	56.4	Thermophilic	Specialized	Facultative
Dehalococcoides CBDB1		NC_007356.1	1.40	47.0	Mesophilic	Multiple	Anaerobic
Roseiflexus RS 1		NC_009523.1	5.80	60.4	Thermophilic	Specialized	Facultative
Cyanothece sp. ATCC 51142	Cyanobacteria	NC_010546.1	5.46	37.9	Mesophilic	Aquatic	Facultative
Nostoc sp. PCC 7120		NC_003272.1	7.20	41.3	Mesophilic	Multiple	Aerobic
Prochlorococcus marinus str. MIT 9211		NC_009976.1	1.70	38.0	Mesophilic	Aquatic	*
Trichodesmium erythraeum IMS101		NC_008312.1	7.75	34.1	Mesophilic	Aquatic	Aerobic
Deinococcus geothermalis DSM 11300	Deinococcus-Thermus	NC_008025.1	3.25	66.5	Mesophilic	Aquatic	Aerobic
Meiothermus ruber DSM 1279		NC_013946.1	3.10	63.4	Thermophilic	Specialized	Aerobic
Thermus thermophilus HB27		NC_005835.1	2.13	69.4	Thermophilic	Specialized	Aerobic
Bdellovibrio bacteriovorus HD100	Deltaproteobacteria	NC_005363.1	3.80	50.6	Mesophilic	Multiple	Aerobic
Desulfotalea psychrophila LSv54		NC_006138.1	3.66	46.6	Psychrophilic	Specialized	Anaerobic
Geobacter sulfurreducens PCA		NC_002939.4	3.80	60.9	Mesophilic	Multiple	Anaerobic
Lawsonia intracellularis PHE/MN1-00		NC_008011.1	1.71	33.1	Mesophilic	Host associated	Facultative
Syntrophus aciditrophicus SB		NC_007759.1	3.18	51.5	Mesophilic	Multiple	Anaerobic
Elusimicrobium minutum Pei191	Elusimicrobia	NC_010644.1	1.64	40.0	Mesophilic	Host associated	Anaerobic
Campylobacter jejuni subsp. jejuni NCTC 11168	Epsilonproteobacteria	NC_002163.1	1.64	30.5	Mesophilic	Multiple	Microaerophilic
Helicobacter hepaticus ATCC 51449		NC_004917.1	1.80	35.9	Mesophilic	Host associated	Aerobic
Helicobacter pylori 26695		NC_000915.1	1.67	38.9	Mesophilic	Host associated	Aerobic
Wolinella succinogenes DSM 1740		NC_005090.1	2.10	48.5	Mesophilic	Host associated	Microaerophilic
Fusobacterium nucleatum subsp. nucleatum ATCC 25586	Fusobacteria	NC_003454.1	2.17	27.2	Mesophilic	Host associated	Anaerobic
Ilyobacter polytropus DSM 2926		NC_014632.1	3.13	34.4	Mesophilic	Multiple	Anaerobic
Leptotrichia buccalis C-1013-b		NC_013192.1	2.47	29.6	Mesophilic	Host associated	Anaerobic
Sebaldella termitidis ATCC 33386		NC_013517.1	4.47	33.4	Mesophilic	Host associated	Anaerobic
Streptobacillus moniliformis DSM 12112		NC_013515.1	1.71	26.3	Mesophilic	Host associated	Microaerophilic
Acinetobacter sp. ADP1	Gammaproteobacteria	NC_005966.1	3.60	40.4	Mesophilic	Multiple	Aerobic
Escherichia coli str. K-12 substr. MG1655		NC_000913.2	4.64	50.8	Mesophilic	Host associated	Facultative
Francisella tularensis subsp. tularensis SCHU S4		NC_006570.2	1.90	32.3	Mesophilic	Multiple	Aerobic
Haemophilus ducreyi 35000HP		NC_002940.2	1.70	38.2	Mesophilic	Host associated	Anaerobic
Azotobacter vinelandii DJ		NC_012560.1	5.40	65.7	Mesophilic	Multiple	Aerobic
Shewanella amazonensis SB2B		NC_008700.1	4.30	53.6	Mesophilic	Multiple	Facultative
Xylella fastidiosa 9a5c		NC_012560.1	5.40	65.7	Mesophilic	Host associated	Aerobic
Denitrovibrio acetiphilus DSM 12809	Other Bacteria	NC_013943.1	3.20	42.5	Mesophilic	Aquatic	Obligate anaerobic

Planctomyces limnophilus DSM 3776	Planctomycetes	NC_014148.1	5.44	53.7	Mesophilic	Aquatic	Aerobic
Rhodopirellula baltica SH 1		NC_005027.1	7.15	55.4	Mesophilic	Aquatic	Aerobic
Borrelia burgdorferi B31	Spirochaetes	NC_001318.1	1.52	28.2	Mesophilic	Host associated	Aerobic
Leptospira borgpetersenii serovar Hardjo-bovis JB197		NC_008510.1	3.88	40.2	Mesophilic	Multiple	Aerobic
Spirochaeta smaragdinae DSM 11293		NC_014364.1	4.70	50.0	Mesophilic	Specialized	Obligate anaerobic
Treponema denticola ATCC 35405		NC_002967.9	2.84	37.9	Mesophilic	Host associated	Anaerobic
Acholeplasma laidlawii PG-8A	Tenericutes	NC_010163.1	1.50	31.9	Mesophilic	Multiple	Facultative
Candidatus Phytoplasma australiense		NC_010544.1	0.88	27.4	Mesophilic	Host associated	Aerobic
Mesoplasma florum L1		NC_006055.1	0.79	27.0	Mesophilic	Host associated	Facultative
Mycoplasma capricolum ATCC 27343		NC_007633.1	1.01	23.8	Mesophilic	Host associated	Facultative
Mycoplasma gallisepticum CA06_2006.052-5-2P		NC_018412.1	0.98	31.6	*	Host associated	Facultative
Mycoplasma mobile 163K		NC_006908.1	0.78	25.0	Mesophilic	Host associated	Facultative
Mycoplasma mycoides SC PG1		NC_005364.2	1.21	24	Mesophilic	Host associated	Facultative
Mycoplasma pulmonis UAB CTIP		NC_002771.1	0.96	26.6	Mesophilic	Host associated	Facultative
Mycoplasma synoviae 53		NC_007294.1	0.79	28.5	Mesophilic	Host associated	Facultative
Onion yellows phytoplasma OY-M		NC_005303.2	0.85	27.8	Mesophilic	Host associated	Aerobic
Ureaplasma parvum serovar 3 ATCC 27815		NC_010503.1	0.75	25.5	Mesophilic	Host associated	Facultative
Ureaplasma urealyticum serovar 10 str. ATCC 33699		NC_011374.1	0.87	25.8	Mesophilic	Host associated	Facultative
Kosmotoga olearia TBF 19.5.1	Thermotogae	NC_012785.1	2.30	41.5	Mesophilic	Aquatic	*
Petrotoga mobilis SJ95		NC_010003.1	2.17	34.1	Thermophilic	Specialized	Anaerobic
Fervidobacterium nodosum Rt17-B1		NC_009718.1	1.95	35.0	Thermophilic	Specialized	Anaerobic
Thermosipho africanus TCF52B		NC_011653.1	2.00	30.8	Hyperthermophilic	Multiple	Anaerobic
Thermotoga lettingae TMO		NC_009828.1	2.14	38.7	Hyperthermophilic	Aquatic	Anaerobic
Thermotoga maritima MSB8		NC_000853.1	1.86	46.2	Hyperthermophilic	Specialized	Anaerobic
Thermotoga naphthophila RKU-10		NC_013642.1	1.80	46.1	Hyperthermophilic	Specialized	Anaerobic

* - Information not available.
