# Supplementary material for: Association of purine asymmetry, strand-biased gene distribution and PolC within Firmicutes and beyond: a new appraisal
Source: BMC Genomics. 2014 Jun 4;15(1):430. doi: 10.1186/1471-2164-15-430 (PMC4070872; doi:10.1186/1471-2164-15-430)
Supplement: Supplementary file 3 — Additional file 3: Figure S1: (L) Cumulative GC-skew (blue lines) and AT-skew (red lines) and (R) purine/pyrimidine skews (black lines) in some model representatives of Trend II organisms. (A) Streptococcus agalactiae NEM316, (B) Acidaminococcus intestini RyC-MR95, (C) Geobacillus kaustophilus HTA426, (D) Veillonella parvula DSM 2008, (E) Thermodesulfobium narugense DSM 14796, (F) Clostridiales genomosp BVAB3 UPII9 5, (G) Acinetobacter sp.ADP1, (H) Candidatus Protochlamydia amoebophila UWE25. (PDF 528 KB) [file 12864_2013_6136_MOESM3_ESM.pdf]

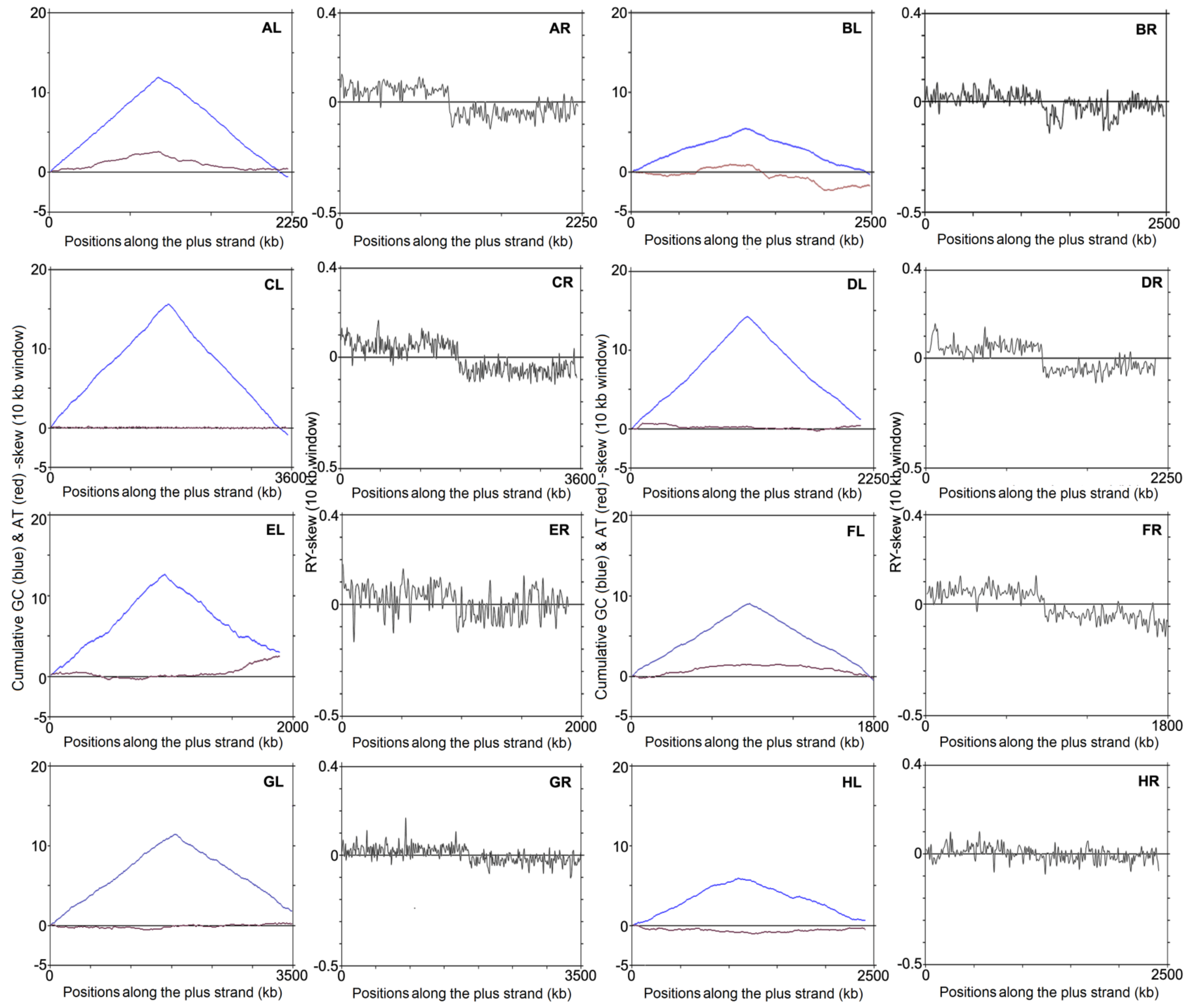

Additional file 3: Figure S1. (L) Cumulative GC-skew (blue lines) and AT-skew (red lines) and (R) purine pyrimidine skews (black lines) in some model representatives of Trend II organisms. (A) *Streptococcus agalactiae NEM316*, (B) *Acidaminococcus intestini RyC-MR95*, (C) *Geobacillus kaustophilus HTA426*, (D) *Veillonella parvula DSM 2008*, (E) *Thermodesulfobium narugense DSM 14796*, (F) *Clostridiales genomosp BVAB3UPII9 5*, (G) *Acinetobacter sp.ADP1*, (H) *Candidatus Protochlamydia amoebophila UWE25*.
