# Supplementary material for: Association of purine asymmetry, strand-biased gene distribution and PolC within Firmicutes and beyond: a new appraisal
Source: BMC Genomics. 2014 Jun 4;15(1):430. doi: 10.1186/1471-2164-15-430 (PMC4070872; doi:10.1186/1471-2164-15-430)
Supplement: Supplementary file 4 — Additional file 4: Figure S2: Trends in individual base usages in Escherichia coli str. K-12 substr. MG1655 for genes encoded by both LeS and LaS. Subscripts are same as in Figure 7. (PDF 688 KB) [file 12864_2013_6136_MOESM4_ESM.pdf]

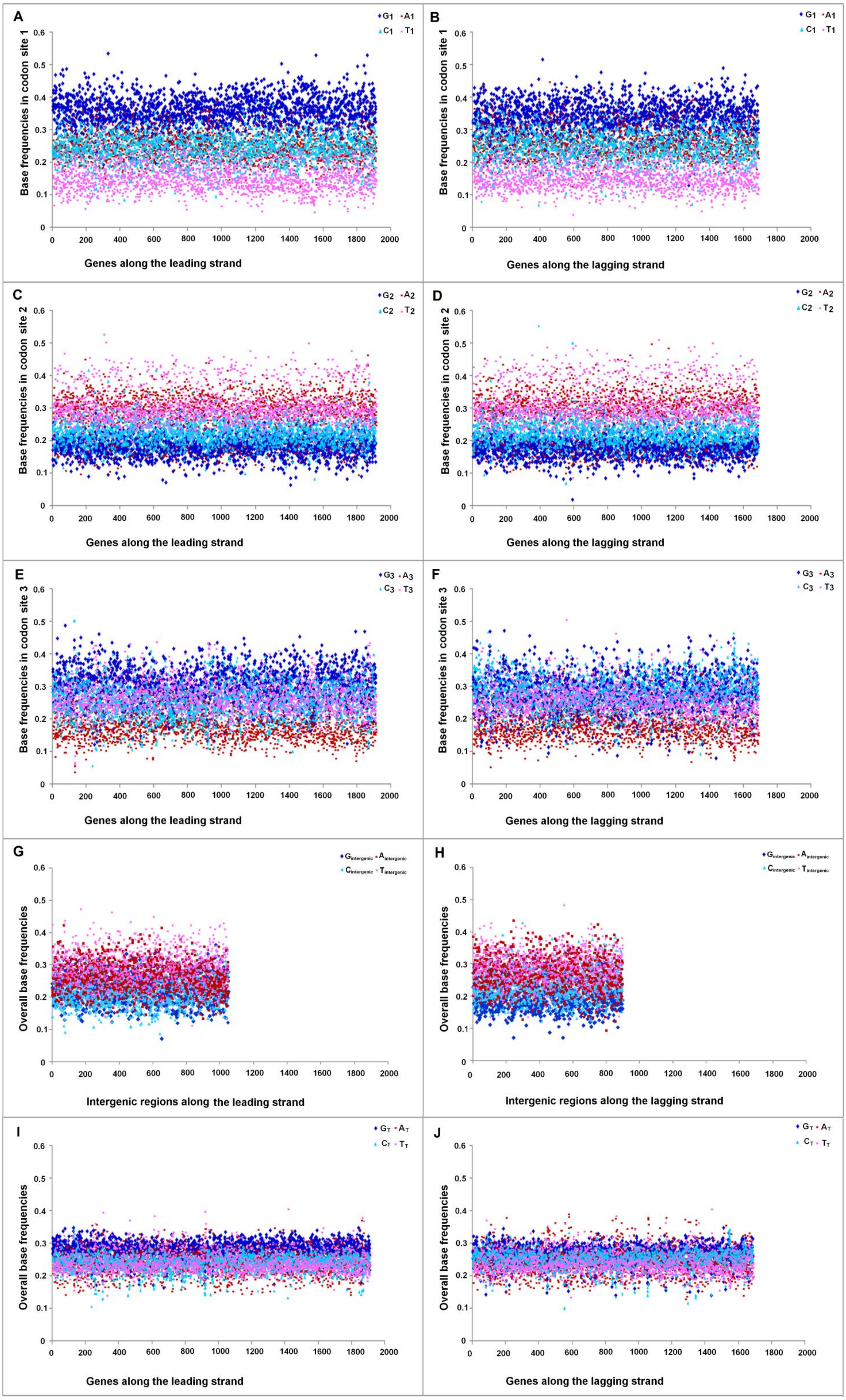

Additional file 4: Figure S2. Trends in individual base usages in *Escherichia coli str. K-12 substr. MG1655* for genes encoded by both LeS and LaS. Subscripts are same as in Figure 7.
