# Supplementary material for: Association of purine asymmetry, strand-biased gene distribution and PolC within Firmicutes and beyond: a new appraisal
Source: BMC Genomics. 2014 Jun 4;15(1):430. doi: 10.1186/1471-2164-15-430 (PMC4070872; doi:10.1186/1471-2164-15-430)
Supplement: Supplementary file 5 — Additional file 5: Figure S3: Trends in individual base usages in Bartonella henselae str.Houston-1 for genes encoded by both leading and lagging strands. Subscripts are same as in Figure 7. (PDF 1 MB) [file 12864_2013_6136_MOESM5_ESM.pdf]

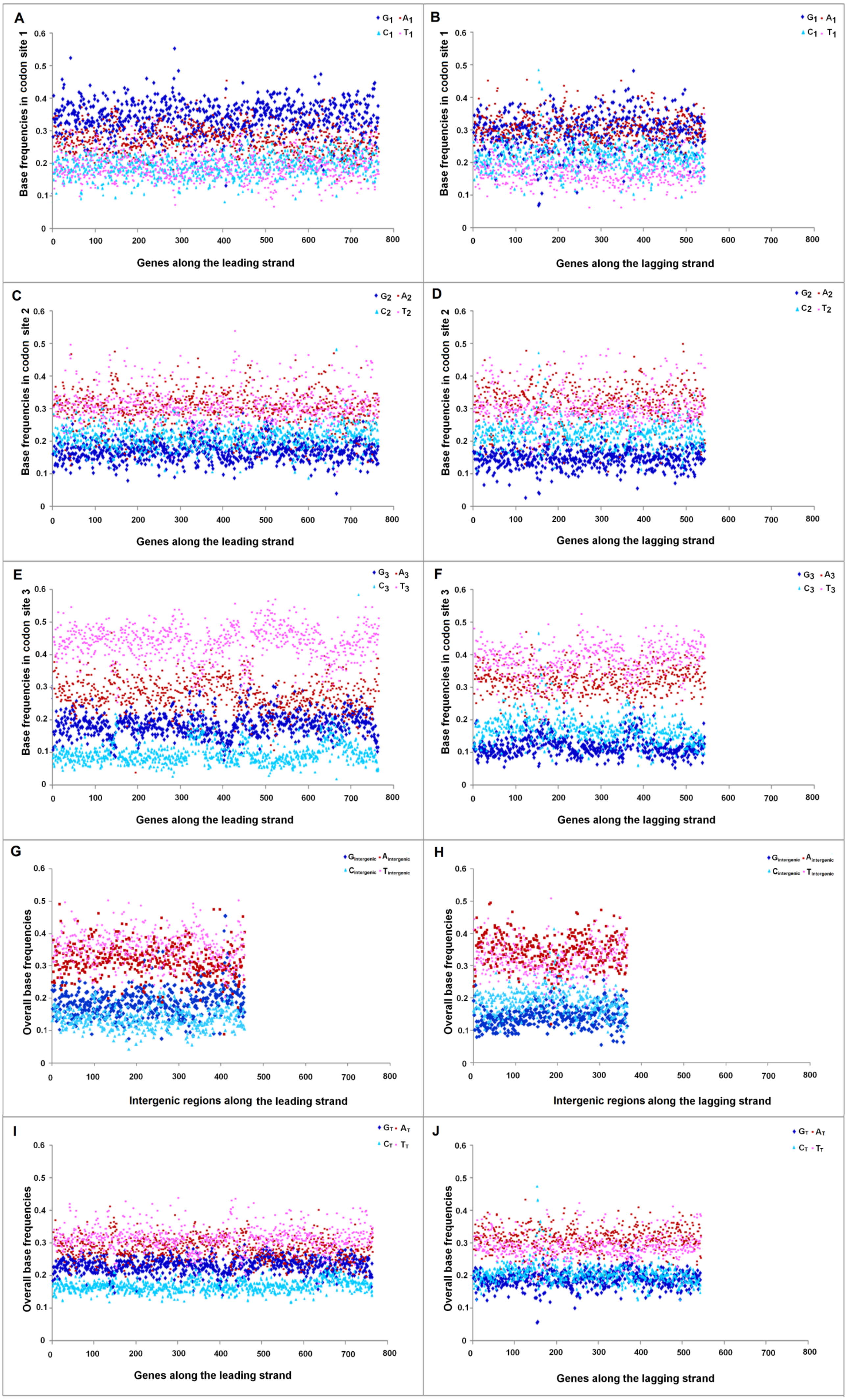

Additional file 5: Figure S3. Trends in individual base usages in *Bartonella henselae str.Houston-1* for genes encoded by both leading and lagging strands. Subscripts are same as in Figure 7.
