# Supplementary material for: Association of purine asymmetry, strand-biased gene distribution and PolC within Firmicutes and beyond: a new appraisal
Source: BMC Genomics. 2014 Jun 4;15(1):430. doi: 10.1186/1471-2164-15-430 (PMC4070872; doi:10.1186/1471-2164-15-430)
Supplement: Supplementary file 6 — Additional file 6: Figure S4: (L) Instantaneous GC-skew (blue lines) and AT-skew (red lines) and (R) Cumulative GC-skew (blue lines) and AT-skew (red lines) in Yerisina pestis strains. (A) Yersinia pestis CO92, (B) Yersinia pestis D106004, (C) Yersinia pestis D106004 (D) Yersinia pestis Antiqua, (E) Yersinia pestis Nepal516, (F) Yersinia pestis KIM 10, (G) Yersinia pestis biovar Microtus 91001, (H) Yersinia pestis Pestoides F. Table S3. Status of combinations (a) – (d) in Y. pestis strains under study. (PDF 1 MB) [file 12864_2013_6136_MOESM6_ESM.pdf]

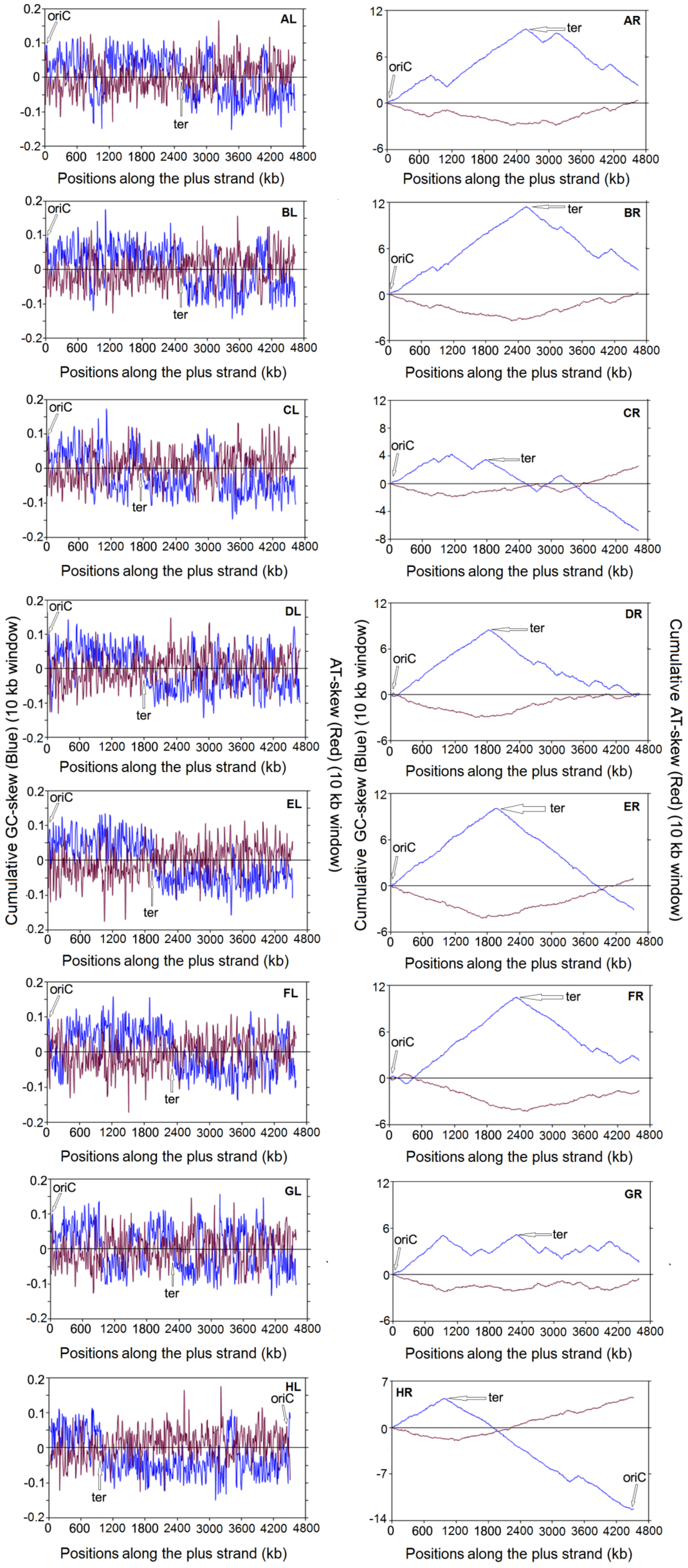

Additional file 6: Figure S4. (L) Instantaneous GC-skew (blue lines) and AT-skew (red lines) and (R) Cumulative GC-skew (blue lines) and AT-skew (red lines) in *Yerisina pestis* strains. (A) *Yersinia pestis CO92*, (B) *Yersinia pestis D106004*, (C) *Yersinia pestis D106004* (D) *Yersinia pestis Antiqua*, (E) *Yersinia pestis Nepal516*, (F) *Yersinia pestis KIM 10*, (G) *Yersinia pestis biovar Microtus 91001*, (H) *Yersinia pestis Pestoides F*.

Additional file 6: Table S3. Status of combinations (a) – (d) in *Y. pestis* strains under study.

Organisms	% of 10 kb segments along LeS with combinations[@]				GC/AT Trends
	**G > C A > T**	**G > C A ≤ T**	**G ≤ C A > T**	**G ≤ C A ≤ T**	
Yersinia pestis CO92	*23.7*	**57.4**	12.3	6.7	IV
Yersinia pestis D106004	*23.1*	**62.7**	9.3	5.0	IV
Yersinia pestis D182038	*22.7*	**54.5**	15.8	6.9	IV
Yersinia pestis Antiqua	*24.0*	**58.7**	11.9	5.3	IV
Yersinia pestis Nepal516	*23.9*	**68.6**	3.1	4.4	IV
Yersinia pestis KIM 10	*22.6*	**60.0**	10.9	6.5	IV
Yersinia pestis biovar Microtus 91001	18.6	**47.7**	*23.9*	9.8	V
Yersinia pestis Pestoides F	*24.4*	**65.9**	5.5	4.2	IV

[@:] Bolds are significant at $p < 0.05$, italics are random.
